# Smart distributed data factory volunteer computing platform for active learning-driven molecular data acquisition

**DOI:** 10.1038/s41598-025-90981-6

**Published:** 2025-02-28

**Authors:** Tsolak Ghukasyan, Vahagn Altunyan, Aram Bughdaryan, Tigran Aghajanyan, Khachik Smbatyan, Garegin A. Papoian, Garik Petrosyan

**Affiliations:** 1 Deep Origin, Yerevan, Armenia; 2 Deep Origin, South San Francisco, USA

**Keywords:** Volunteer computing, Active learning, Conformational energy, Machine learning, Graph neural networks, Molecular conformation, Software, Scientific data, Virtual drug screening

## Abstract

This paper presents the smart distributed data factory (SDDF), an AI-driven distributed computing platform designed to address challenges in drug discovery by creating comprehensive datasets of molecular conformations and their properties. SDDF uses volunteer computing, leveraging the processing power of personal computers worldwide to accelerate quantum chemistry (DFT) calculations. To tackle the vast chemical space and limited high-quality data, SDDF employs an ensemble of machine learning (ML) models to predict molecular properties and selectively choose the most challenging data points for further DFT calculations. The platform also generates new molecular conformations using molecular dynamics with the forces derived from these models. SDDF makes several contributions: the volunteer computing platform for DFT calculations; an active learning framework for constructing a dataset of molecular conformations; a large public dataset of diverse ENAMINE molecules with calculated energies; an ensemble of ML models for accurate energy prediction. The energy dataset was generated to validate the SDDF approach of reducing the need for extensive calculations. With its strict scaffold split, the dataset can be used for training and benchmarking energy models. By combining active learning, distributed computing, and quantum chemistry, SDDF offers a scalable, cost-effective solution for developing accurate molecular models and ultimately accelerating drug discovery.

**Supplementary Information:**

The online version contains supplementary material available at 10.1038/s41598-025-90981-6.

## Introduction

Machine learning (ML) techniques have reshaped many data-rich fields, such as computer vision and natural language processing, demonstrating that increasing data size can significantly impact model accuracy. This success has sparked interest in applying ML to drug discovery, where data-driven modeling could revolutionize molecular design and property prediction. However, the vast chemical space (“drug-like” chemical space is estimated at the order of 10^60^ molecules^1^) poses a unique challenge: for many critical problems, the available data is limited and often not optimized for machine learning tasks.

Existing molecular datasets, while valuable, present several limitations. Many were not originally designed for training data-hungry ML algorithms but were instead compiled from wet lab experiment reports or created for computational chemistry benchmarks. As a result, their data distribution, coverage of chemical space, and level of theory may not align optimally with the specific requirements of ML methods in drug discovery. For instance, the QM9 dataset^2^, widely used for training and benchmarking conformational energy prediction models, provides only one conformation per molecule and lacks scaffold diversity. Furthermore, many of its test set scaffolds (60–99% depending on the split) are present in the training set, indicating data leakage. Similar issues of restricted chemical spaces, potential biases, or limited diversity are seen in other datasets like ANI-1^3–5^, NablaDFT^6^, and MPCONF196^7^. All of these datasets provide at best scaffold-based train-test split (without any additional similarity constraints), and also contain mostly smaller molecules relative to what is required for drug discovery projects. Gasteiger et al.^8^ demonstrated the discrepancy in model development decisions, originating from training datasets, and highlighted the importance of dataset diversity and size. Using diverse and large compound libraries of drug-like molecules such as ENAMINE^9^ would address most of the aforementioned issues.

The mismatch between existing datasets’ intended use and the emerging needs of modern machine learning techniques extends beyond specific properties like conformational energies. This limitation can hinder the development of robust and generalizable models because the energy landscape of small molecules plays an important role in many other properties, such as binding affinity, solubility, and synthesizability. Therefore, creating high-quality data and methods for energy landscape modeling can be beneficial for various tasks, encompassing a wide range of molecular characteristics crucial for drug design.

Density Functional Theory (DFT) calculations have emerged as a powerful tool for accurately estimating molecular geometries, energies, and various other properties. However, the computational cost associated with these quantum mechanical methods can be prohibitive, especially for large-scale datasets. On the other hand, while machine learning techniques have shown great promise in rapidly predicting molecular properties, their performance is heavily dependent on the quality and diversity of the training data.

Distributed computing has proven to be a powerful approach for addressing computationally demanding problems, particularly in scientific fields^10,11^. Projects like Folding@home^12^, which leverages the collective power of volunteers to simulate protein folding, have shown the potential of distributed computing to tackle large-scale biochemical problems. Similarly, Drugit^13^, a citizen science initiative, highlights how human intuition can enhance small molecule design by engaging non-experts in collaborative problem-solving. Building on these methodologies, our work integrates the principles of distributed computing with active learning to address the challenges of dataset creation for machine learning in drug discovery.

In this paper, we introduce “Smart Distributed Data Factory,” a framework that leverages active learning and volunteer-based distributed computing to construct a comprehensive, high-quality dataset of molecular conformations with their DFT-calculated properties. With the help of volunteer computing we hope to accelerate these DFT calculations by harnessing the collective processing power of numerous personal computers worldwide. Our approach uses an ensemble of diverse machine learning models to predict conformational energies and strategically selects the most challenging instances for DFT calculations. This targeted selection process ensures that the most valuable data points are prioritized, leading to efficient data acquisition and improved model performance.

Key contributions of our work include:

1. A highly scalable and AI-powered distributed computing platform that engages volunteers worldwide to perform quantum mechanical calculations, accelerating dataset growth.
2. A novel active learning framework tailored to construct a dataset of molecular conformations with DFT-calculated properties, featuring an ensemble of models with different architectures to enhance prediction diversity and sampling efficiency.
3. An ensemble of machine learning models for accurate prediction of molecule conformational energies.
4. A large and continuously growing public dataset of diverse molecules sampled from the ENAMINE database, that serves as both a training resource and a rigorous benchmark for energy prediction models.

Our methodology offers a scalable and cost-effective solution for building comprehensive datasets tailored to the specific needs of molecular modeling applications. By combining the power of machine learning, distributed computing and quantum chemistry calculations, we aim to enhance the development of accurate and reliable computational models for conformational analysis, ultimately accelerating the discovery and design of novel molecules, particularly for drug development.

The datasets generated in this work are released at www.zenodo.org/records/14008357. The developed energy prediction models and inference code are freely available at www.github.com/deeporiginbio/smartdatafactory-experiments.

## Results

We present Smart Distributed Data Factory (SDDF), a distributed computing platform designed for labeled molecule dataset generation using volunteer user machines. It supports a wide range of structure-based molecular property prediction tasks. At its core, SDDF employs an ensemble-based active learning strategy, utilizing models with different architectures to intelligently select the most informative instances from a vast pool of unlabeled data. Having several different architectures is important as the data generation process will select the best architecture for given data size and distribution.

In this section we describe the key elements of the SDDF platform in more detail, including the demonstration of the platform’s use for the creation of a conformational energy dataset. In addition to the dataset we provide a subset of it as a benchmark with a strict scaffold and similarity split to training, validation and test sets (denoted as SDDF train, SDDF validation, and SDDF test, respectively). We anticipate that SDDF will be used for the creation of molecule datasets not just for energy predictions, but also other structure-based properties.

### Smart distributed data factory volunteer computing platform

The SDDF platform provides a website (https://sddfactory.cloud*)* where volunteers can sign up and receive molecular conformations for DFT calculations on their personal computers. Each calculation task consists of a single conformation of a molecule and a property specifier indicating a set of properties to calculate. For a targeted property and an average-sized molecule, a single-core machine is expected to calculate the property in about 10 min. The result of each task is a dictionary with property names as keys and respective calculated values.

Users can select the projects to which they want to contribute calculations, and they will receive computational tasks only from those projects. Otherwise, the platform assigns tasks from randomly selected projects.

### Performance benchmarks

To evaluate the performance of SDDF, we conducted several benchmarks. The following aspects were considered:

- *Message broker reliability* The file-based message broker ensures high reliability and system uptime without the overhead of managing third-party services. Tests indicate that it can handle thousands of messages per second with minimal latency.
- *Resource utilization* Volunteers’ machines use approximately 50% of their computational resources, with each task running on around three threads by default to balance performance and reliability.
- *Task completion time* For a targeted property and an average-sized molecule (~ 25 heavy atoms), a single-core (2.4 GHZ) machine completes a calculation task in about 10 min.

By carefully considering these factors, SDDF provides a robust platform for distributed computing, capable of generating high-quality labeled datasets for molecular property prediction.

### Creating projects for data labeling via DFT

The platform allows to set up different dataset creation projects. For each project, the creator must provide the set of input molecules, and set the parameters for the computational tasks (such as minimum and maximum examples per task, DFT configuration, results verification threshold, etc.). The labeled datasets for all projects created via the platform will be publicly available under CC BY 4.0 license.

### Task definition and volume estimation

We define each computational task as a single molecular conformation for which the user has to calculate the target property using DFT. The volume of each task is estimated based on the number of its conformation’s atoms. The conformations are selected from a pool of unlabeled examples provided by the project’s administrator.

The user side receives the task as an internal gRPC structure, which contains task type (indicating properties needed to calculate), identifier and task content which is a MOL block representation of desired molecule conformation. After the required calculations are completed, the results are sent to the server side encapsulated in internal gRPC structure containing task type, identifier and task results represented as JSON string.

### Setup and minimal system requirements

The volunteer computing software can be installed on machines with Linux or MacOS systems (support for Windows will be added in near future). It is set up on the user machines as a Python module. The user needs to specify their credentials and other parameters specified in their accounts in the config file to collect points and climb up in the leaderboard. Then by running the script in background, the machine starts to periodically fetch tasks from the server, do computations and return results.

To be able to run the computational tasks in a reasonable time, the user machine should be able to allocate 8GB RAM and 10 GB disk space (for storing the temporary files generated during DFT calculations). All computations on the volunteer machines are CPU-only.

### Active-learning based data sampling

**Fig. 1:**
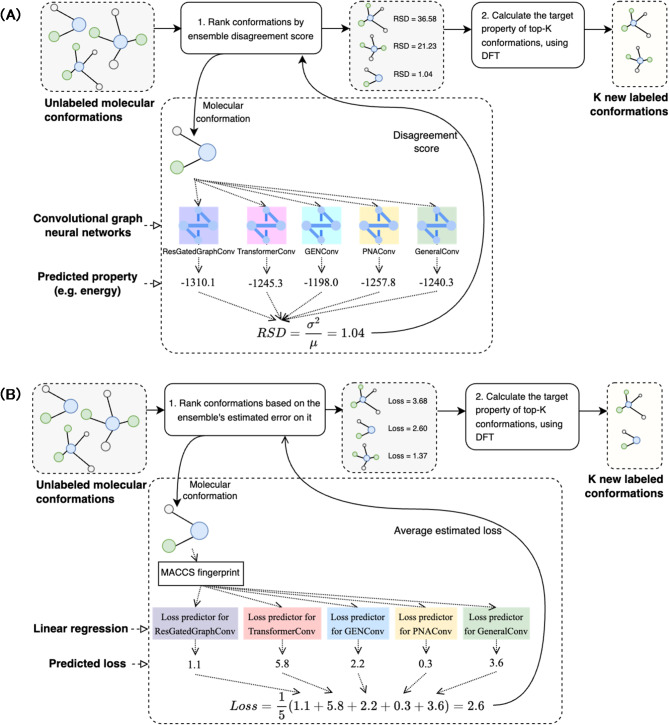
The labeling workflow of the SDDF datasets based on different sampling algorithms: ensemble models’ disagreement-based approach (**A**) and ML-based loss prediction approach (**B**). (**A**) Illustrates the ensemble models’ disagreement-based conformation sampling workflow, using the relative standard deviation of model predictions as the disagreement score. (**B**) Illustrates the ML-based conformation sampling workflow, using the input molecule’s MACCS fingerprint and multi-layer perceptron to predict the ensemble models’ error on the input.

SDDF implements an active learning framework to select molecules for labeling and addition to the dataset. The framework iteratively samples molecules from a large database in random fashion and generates multiple conformations for each molecule using RDKit^14,15^ and MD. At each iteration, a fraction of the generated conformations is selected and labeled, after which they are added to the dataset. The selection is performed based on an ensemble of ML models, which are used to determine the most challenging conformations among the generated set of conformations. The target property of the selected most challenging conformations is calculated using DFT. In addition, the selected conformations are used as initial points for MD calculations and the intermediate structures from the calculated trajectories are also labeled via DFT. All newly labeled examples are incorporated into the dataset, and used to re-train the ML ensemble. The workflow for the molecule conformational energy dataset creation is illustrated in Fig. 1.

In order to train the ML ensemble, our platform labels a small initial dataset of randomly selected conformations, and then its constituent models are re-trained after each iteration of data selection and labeling.

### Ensemble of predictors

We use an ensemble of ML-based predictors where each predictor is a model trained separately as a regression problem that gets the molecular conformation graph as input and outputs an energy prediction. The nodes of the input graph are the molecule’s atoms and its adjacency matrix is constructed based on the bonds and distances between atoms (we considered an atom pair as adjacent if they have a bond or their distance is below a threshold value).

**Fig. 2:**
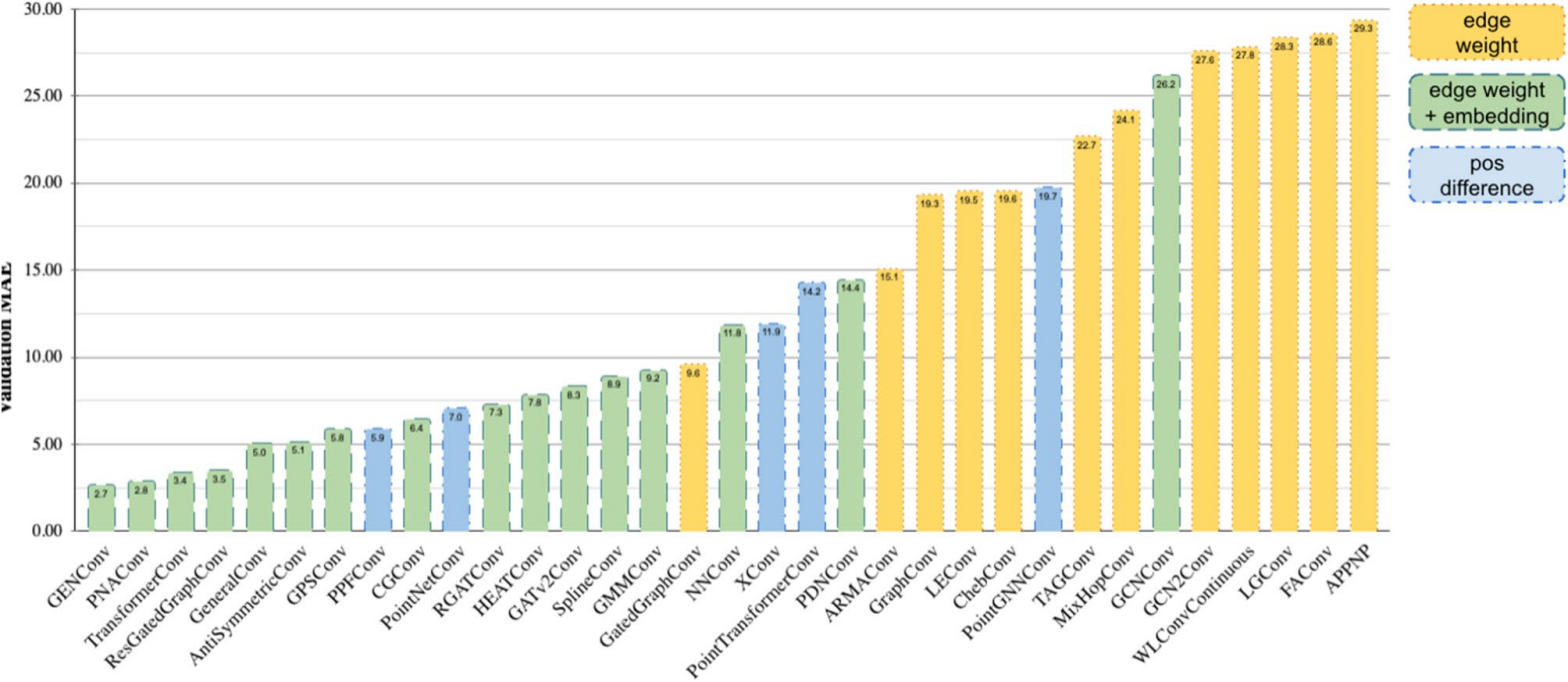
Performance evaluation of GCNN and Point Cloud models on the validation set. Validation MAE is reported in kcal x mol^−1^. The models are stylized differently based on the method of incorporating the structure’s 3d information: using atom distance-based weights for the edges; using distance-based weights and edge embeddings; using the difference of atom position vectors).

We performed initial model selection by training and evaluating 33 different graph convolutional neural network (GCNN) and Point Cloud architectures implemented in PyTorch Geometric^16^ for the conformational energy prediction task. Based on the evaluation results (Fig. 2) we selected the 5 models with the best mean absolute error (MAE) scores on the validation set: GeneralConv^17^, PNAConv^18^, GENConv^19^, TransformerConv^20^ and ResGatedGraphConv^21^ models, as implemented in PyTorch Geometric. We further improved the models’ performance by employing Point Pair Features^22^ for bonded atoms. The description of the models, their selection and hyperparameter tuning processes is described in more detail in the Methods section and Supplementary B.

The decision to use different architectures is based on the assumption that, as the data changes, the effectiveness of the models may also vary. Thus, having a variety of models allows the ensemble to be more robust to distribution shifts in the data. Additionally, we plan to introduce a model selection feature to the platform in the future, where novel architectures could be added to the ensemble if they demonstrate better test scores compared to the existing ones. It is important to note that this selection process will remain dynamic; some models may be dropped if their relative performance declines with more data, while novel architectures will be introduced in their place.

### Selection of molecular conformations

We investigated different strategies for the selection of molecular conformations for further labeling, with the final approach in our software utilizing machine learning-based predictors. These predictors are linear regression models that estimate the corresponding ensemble models’ error for a given molecule. Each regression model takes the MACCS^23^ fingerprint of the molecule as input and is trained on the same dataset as the ensemble. The training process employs a regression approach, predicting the MAE between the ensemble model’s prediction and the actual conformational energy. A high predicted error value indicates a challenging and thus valuable instance for data acquisition. The conformations are ranked based on this error (from highest to lowest), and the highest ranked are selected for DFT calculations and subsequent inclusion in the dataset. The predictor’s relatively simple architecture and its independence from 3D structural information enable rapid error estimation across a large molecular dataset, offering an efficient alternative for assessing model uncertainty without relying on variance-based methods.

Additionally, we explored an earlier strategy based on a disagreement score calculated from the predictions of the ML ensemble. For SDDF, this score is based on the relative standard deviation of model predictions, quantifying the uncertainty or conflict among the ensemble’s predictions for a given conformation. While this method has been validated in several previously published works^24,25^, the machine learning-based approach ultimately provided a more efficient solution in our final implementation.

To evaluate the effectiveness of the proposed data sampling strategies, we conducted a series of experiments, where we viewed the molecule selection problem in the data stream setup, inspired by data stream active learning techniques^26^. To support our choice of an ensemble with heterogeneous architectures, we investigated four sampling configurations: (i) random sample selection (RAND); (ii) using the relative standard deviation of the predictions of an ensemble of five identically architected GENConv models with different random initializations (x5GENConv); (iii) using the relative standard deviation of the predictions of an ensemble of five different architectural models (SDDF-VAR); (iv) using the model-predicted error of an ensemble of five different architectural models (SDDF-LOSSFN).

**Fig. 3:**
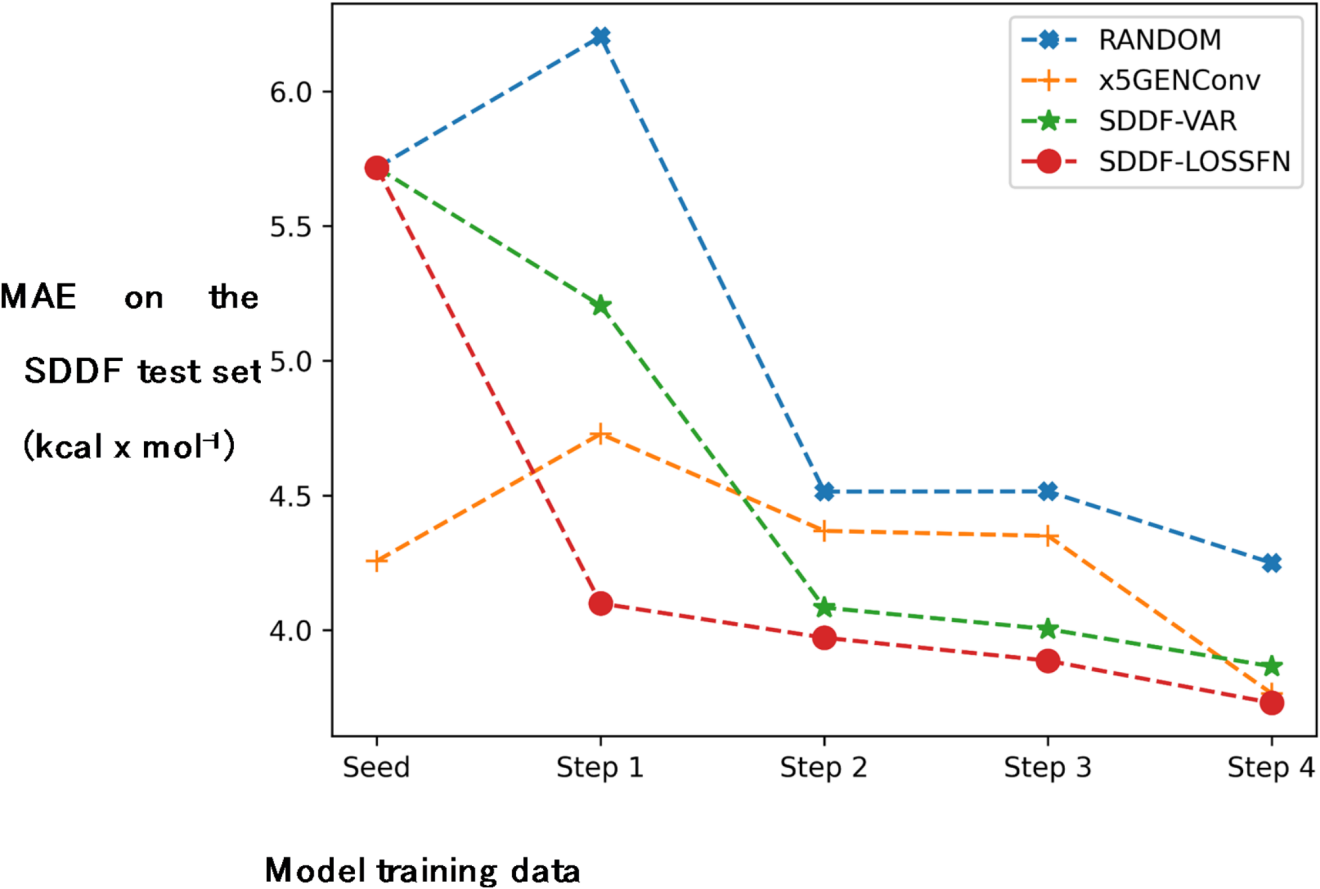
Comparison of data selection strategies. SDDF-VAR is the sampling strategy using the variance-based disagreement score of a heterogeneous ensemble of ML models; SDDF-LOSSFN is the sampling strategy using the predicted model error for a heterogeneous ensemble of ML models; x5GENConv denotes the sampling strategy using the variance-based disagreement score of a homogenous ensemble of ML models (5 GENConv with different initializations); RANDOM denotes random sampling. We report the MAE (kcal x mol^−1^) on the SDDF test set.

Based on the results (Fig. 3), we can conclude that using ensemble-based sampling is more effective than random selection. Among the ensemble-based approaches, the most rapid test performance improvement was achieved by the loss prediction-based sampling method. The results also demonstrate the importance of diverse architectures in the ensemble configuration, as SDDF ensemble displayed the most rapid improvement. x5GENConv’s use of varied initializations within the same architecture also allowed for a consistent but slower improvement, highlighting the benefit of using the disagreement of more diverse models. The performance of RANDOM served as a baseline. The experiment results support the hypothesis that ensembles, particularly heterogeneous architectures as in SDDF, can enhance the effectiveness of data selection in active learning, leading to more rapid and potent improvements in model performance compared to homogeneous ensembles or random selection strategies.

### Molecular dynamics-based data sampling

In addition to the active learning-based sampling of new data points, we also employ the top-performing models of its ensemble to perform Langevin MD and generate new conformations for labeling. This approach uses a randomly selected small subset of the existing set of conformations, and generates the MD trajectories for each conformation in the subset. For the atoms in the generated conformations we obtain the forces using the gradient of the SDDF ensemble model’s predicted energies. For each atom, we calculate the pairwise cosine similarities of the gradients and select the minimum similarity as a confidence score of the ensemble’s prediction. Then, we compute the average of these atomwise confidence scores for each conformation (Eq. (1)), and select the conformations with the lowest average confidence for final labeling via DFT.

1 $$\:Confidence\:score\:=\:\frac{1}{{N}_{atoms}}{{\sum\:}_{i=1}^{{N}_{atoms}}}^{}min\left(\right\{cos\angle\:({{F}_{q}}^{i},{{F}_{k}}^{i})|\:q,k\in\:Ensemble,\:q\ne\:k\}),$$

where $\:{{F}_{m}}^{i}\:$is the gradient of the $\:m$-th model’s predicted energy with respect to the $\:i$-th atom’s position.

We evaluated the effectiveness of the MD-based conformation generation and sampling in the task of energy minimization. The experiment was performed with the assumption that Langevin MD should generally lead to a stable conformation that is near a local minimum of the energy landscape because it is guided by the negative gradients of the potential energy. We randomly selected around 400 conformations from the SDDF test set that were not optimized using MMFF94, and then used SDDF ensembles to generate MD trajectories for these test conformations. We did not include MMFF94-optimized conformations in this test because our assumption about energy minimization is more likely to hold for non-equilibrium conformations compared to force-field optimized conformations.

MD was performed using 3 different ensembles for the prediction of forces: (i) SDDF ensemble trained on the full SDDF train set, (ii) SDDF + 50k-RANDOM ensemble trained on the full SDDF train set and randomly sampled 50k new conformations outside the train the set, (iii) SDDF + 50k-MD ensemble trained on the full SDDF train set and additional 50k conformations generated via MD sampling based on 10k random examples from the SDDF train set. Thus, we obtained 3 new sets of conformations and labeled them using DFT. We calculated the difference in the energies for the conformations in the 3 generated conformation sets and the original set to estimate the impact of additional data on the ensemble’s ability to guide the molecules to a lower-energy conformation.

The experiment’s results are provided in Table 1. The calculated correlation coefficient is Spearman’s rank correlation between the differences of predicted energies and true differences of the energies of the initial and final conformations. We also calculated the portion of conformations where the energy decreased after MD, and the average difference between resulting conformations’ energies and the original energies. The results show that the addition of training examples generated via MD-based sampling has a much more positive impact on the ensemble’s ability to do MD than the addition of randomly sampled train examples of the same number.

**Table 1:** Comparison of SDDF ensembles in the conformational energy optimization task.

	SDDF	SDDF + 50k-RANDOM	SDDF + 50k-MD
Spearman correlation of the differences	0.409	0.463	0.708
Success rate	13.33%	13.56%	38.16%
Minimized energy average (Hartree)	0.094	0.099	0.005

Spearman’s rank correlation is calculated between the differences of predicted energies and true differences of the energies of the final and initial conformations (i.e. between the values of $\:SDDF\left({x}_{final}\right)-SDDF\left({x}_{inital}\right)$ and $\:DF{T}_{\omega\:B97X/6-31G\left(d\right)}\left({x}_{final}\right)-DF{T}_{\omega\:B97X/6-31G\left(d\right)}\left({x}_{initial}\right)$ for each test conformation $\:x$, where $\:SDDF$ denotes the energy prediction ensemble). Success rate is measured as the portion of original conformations for which the energy was minimized during the MD. The energy difference is measured in Hartree units.

### Conformational energy datasets and machine learning ensemble

#### Conformational energy dataset and benchmark

Smart Distributed Data Factory (SDDF) relies on a relatively small starting dataset of labeled examples, which are used to train its ensemble’s initial models. For the conformational energy prediction task, we newly created and published a dataset of 2.17 M molecular conformations with DFT-calculated energy labels. The molecular conformations were generated from ENAMINE molecules, using Python’s RDKit toolkit with different random seeds. DFT calculations were performed through Python’s Psi4 toolkit, using the same theory level as ANI datasets: ωB97x^27^ density functional and the 6–31 G(d) basis set^28^. We selected the REAL database of ENAMINE as the source of our examples, because it is a pivotal and extremely relevant collection of synthetically possible molecules used for drug discovery and development projects.

Our full labeled dataset contains 2,170,553 conformations, including 535,338 that were generated using RDKit, another 1,151,936 that were generated using RDKit and optimized with the MMFF94 force field, and 483,279 other conformations generated using MD on RDKit conformations.

In addition, we release a subset of the dataset for training and benchmarking energy prediction machine learning models (the statistics of the split are available in Supplementary C, Table S12). We applied a strict scaffold split to our training, validation and test sets (denoted as SDDF train, SDDF validation, and SDDF test, respectively), so that the maximum Tanimoto similarity between any test and train scaffolds did not exceed 0.7. In this work, we determine the scaffolds using the RDKit implementation of Bemis-Murcko framework^29^.

Apart from using ENAMINE as the source of the molecules, the decision to use a new dataset for the energy prediction task was motivated by other important factors as well: the lack of either conformational or scaffold diversity (Supplementary C, Table S12), the relatively small size of the molecules in the existing datasets (Supplementary C, Fig. S9), or the presence of train test leakage. For example, the QM9 dataset, which is often used as a benchmark, does not provide more than a single conformation for each molecule, and at the same time lacks scaffold diversity (the overall ratio of unique scaffolds to total examples is less than 2%). In addition, more than 60% of the test set scaffolds (Cormorant dataset split^30^) are also present in the train set, indicating leakage from that set.

### Comparison with ANI-2x

Using the architectures selected for the active learning framework, we trained conformational energy prediction models using SDDF training and validation sets. Alongside the datasets, we also publish these models.

In order to validate the effectiveness of the selected model architectures in the energy prediction task, we compared their performance with the ANI-2x ensemble^31^. Table 2 provides the evaluation results of the models on the SDDF test set. We used the TorchANI toolkit (version 2.2.4)^32^ to perform the inference for ANI-2x. Our ensemble and the majority of its individual models outperformed the ANI-2x ensemble in RMSE and MAE metrics.

**Table 2:** Performance of ANI-2x and SDDF ensemble models on our SDDF test’s subset without bromine atoms (the values in the brackets indicate the score on the full test set, including examples with bromine atoms).

Model		Test RMSE (kcal x mol^-1^)		Test MAE (kcal x mol^-1^)	
SDDF	PNAConv	3.38	(3.35)	2.06	(2.06)
	ResGatedGraphConv	3.42	(3.39)	2.24	(2.25)
	GENConv	3.60	(3.58)	2.23	(2.25)
	GeneralConv	5.97	(6.02)	4.08	(4.10)
	TransformerConv	7.34	(7.31)	5.11	(5.12)
	Ensemble{PNAConv, ResGatedGraphConv, GENConv}	**2.92**	**(2.89)**	**1.82**	**(1.83)**
	Ensemble{all}	3.54	(3.52)	2.30	(2.31)
ANI-2x*		5.07	–	2.84	–

*Does not support Bromine.

Significant values are in bold.

**Fig. 4:**
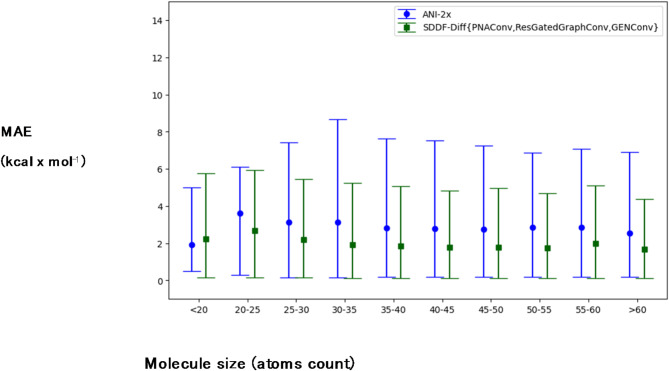
Comparison of ANI-2x and SDDF models’ SDDF test set MAE (kcal x mol^−1^) based on molecule size. For SDDF we report the result of the ensemble of the 3 best-performing models. The displayed ranges indicate the 0.05–0.95 quantiles of the prediction errors for that subset of molecules.

We additionally analyzed the dependence of the models’ performance on the size of test molecules (Fig. 4). The ANI-2x ensemble shows noticeably higher MAE on molecules outside its train data distribution (> 25 atoms), while the MAE of the SDDF ensemble remained relatively more stable across different molecule sizes.

## Discussion

In this paper, we introduced Smart Distributed Data Factory, a framework for generating labeled datasets in computational chemistry. Our ensemble-based active learning strategy, which estimates the error of machine learning models on individual instances, efficiently identifies molecular conformations where high prediction errors are expected, thus prioritizing those instances for DFT calculations.

The SDDF framework integrates two essential components: an ensemble of machine learning models for data selection and a volunteer computing platform for performing DFT calculations. The ensemble consists of five architecturally distinct graph neural networks, which improves its ability to capture a diverse range of molecular complexities and identify instances likely to result in higher errors. This approach ensures that the most challenging and uncertain data points are selected for labeling. Simultaneously, the volunteer computing platform distributes computational tasks to volunteers globally, enabling scalable and distributed data labeling, which significantly enhances the framework’s capacity.

By applying these methods, we created a dataset of molecular conformations along with their DFT-calculated energies. This dataset serves as both a valuable training resource and a benchmark for evaluating energy prediction models. By focusing on instances where models estimate higher prediction errors, the active learning strategy ensures that the dataset is rich in challenging examples, enhancing the overall quality of the training data. These datasets and models trained on them provide a reference in conformer generation for identifying the most stable 3D structures, essential for downstream analyses such as property prediction. In protein-ligand docking, our models can be used for efficient sampling of ligand conformations, enabling more precise binding pose predictions. While virtual screening benefits from these enhanced predictions to rapidly screen chemical libraries, the models are also valuable in lead optimization, where they help assess the impact of structural modifications on binding and guide the design of improved analogs. Moreover, these models can be leveraged as ML-based force fields in molecular dynamics simulations, offering potential energy surfaces for exploration of conformational landscapes and dynamical behaviors.

More generally, the SDDF platform enables the generation of datasets with a wide range of applications. These datasets are valuable for developing molecular property predictors, training and evaluation of conformer generation methods, and conducting crystal structure-related studies, such as crystal polymorphism analysis. For example, DFT calculations can be used to determine molecular geometries corresponding to local energy minima on the potential energy surface, providing optimized and physically meaningful structures for analyses and modeling. They are also crucial for structure-based virtual screening and docking pose prediction, where quantum-derived properties can improve accuracy. For instance, DFT-derived partial charges can be highly valuable input features for models that predict binding affinities, solubilities, and other key properties. Most importantly, these datasets can be used to pre-train structure-based molecular embedding models that learn the underlying energy landscape of molecules. We believe that this capability has the potential to significantly enhance performance across a range of downstream computational tasks, supporting advances in structure-based molecular research.

However, several challenges remain. The scalability of the volunteer computing platform is inherently dependent on participant availability and resource distribution, which may result in uneven computational capacity. Sustaining volunteer engagement and optimizing task distribution algorithms are areas for future improvement. In addition, the platform currently provides computational projects for only conformational energy and atomic charges calculations. In the future, we plan to add projects for the calculation of other properties. Furthermore, while the ensemble of graph neural networks has proven effective, refining active learning strategies and incorporating additional architectures could also improve the framework’s performance. This includes transitioning from fingerprint-based linear regression to more complex methods for predicting the ensemble’s error, such as using 3D structure-sensitive models (for example, GCNNs). Lastly, while the ENAMINE Real database provides a large and synthetically accessible pool of molecules, it does not inherently ensure comprehensive diversity. In future work, we plan to integrate additional source databases which, combined with our active learning-based iterative selection method, will enhance the diversity of the generated datasets.

Regarding the tradeoff between cloud-based and volunteer computing solutions, using our estimates based on mean runtime data, the current dataset generation (requiring approximately 1.5 billion CPU seconds) could be completed on a single high-performance machine (e.g., an AMD EPYC™ 9654 core) in about six months. At a monthly cost of $8,000–$9,000, this would total approximately $50,000. Scaling up to tens of millions of molecules would likely cost into the millions of dollars if done exclusively through commercial cloud or bare-metal resources, particularly when more computationally intensive DFT methods or additional properties (such as RESP charges) are included. The use of spot instances, such as those provided by Google Cloud Platform (GCP) or Amazon Web Services, offers a potentially more cost-effective alternative for reducing costs compared to traditional on-demand pricing. Based on our benchmarking, GCP provides approximately a 65% discount on suitable machines, such as high-memory N4 instances. In a favorable scenario, we believe this approach can reduce the overall costs by a factor of about two. However, several tradeoffs accompany the use of spot instances. First, we observed a 20% increase in calculation time on spot instances compared to their on-demand counterparts. Second, per CPU storage costs escalate because each node must be able to independently process any molecule from the dataset to prevent tool failures and duplicated expenses. Larger spot instances can mitigate this issue by ensuring balanced resource utilization and reducing idle resources, though they are also more likely to be preempted. Finally, orchestrating the workflow to handle machine availability introduces additional complexity and overhead.

Both preemptible cloud computing and volunteer computing rely on “opportunistic” resources, but volunteer computing adopts a fundamentally different paradigm. It emphasizes large-scale scientific collaboration rather than reliance on financial investment to reduce computational time. Volunteer computing leverages the collective contributions of a broad community to achieve computational throughput levels that may surpass what individual institutions can feasibly support. This approach fosters broader engagement in the research process, enabling more extensive and ambitious scientific investigations than would be possible using traditional, centralized methods.

In conclusion, SDDF demonstrates the potential of combining active learning with distributed computing for efficient dataset creation in computational chemistry. By focusing on instances with high error estimates, this framework contributes to the development of more accurate and reliable models for molecular property prediction.

## Methods

### Smart distributed data factory system architecture

**Fig. 5:**
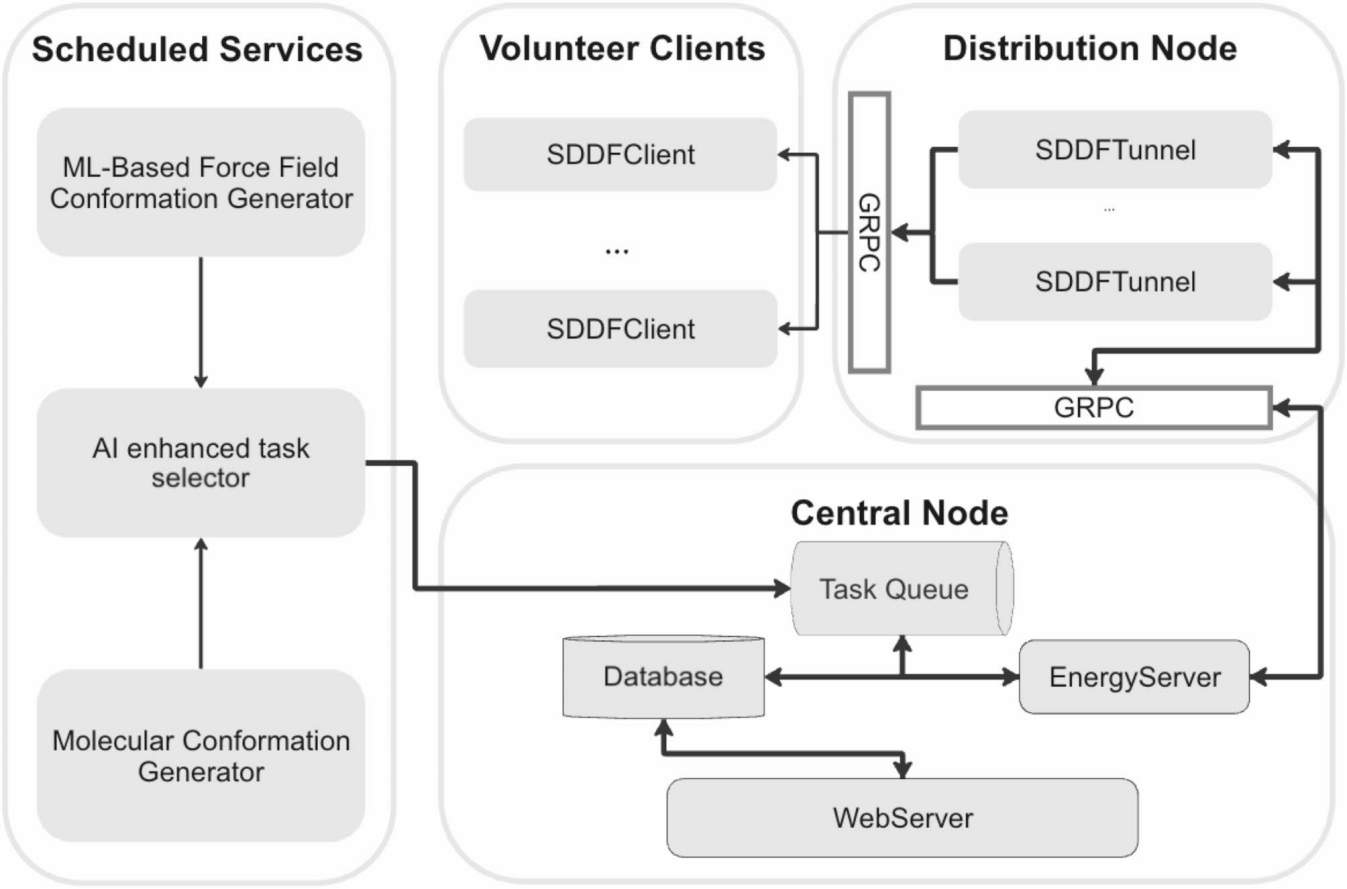
The architecture of the distributed computing system.

The main components of the SDDF system implementation are (illustrated in Fig. 5):

- Central Node:
  - Task Queue: Receives tasks from the Task Publisher and manages the queue of tasks to be processed.
  - Database: Stores data necessary for task processing and system management.
  - SDDF Server: Responsible for forming computational tasks and their distribution. Communicates with the Central Node for data exchange via gRPC.
  - Web Server: Hosts the website and handles interactions with external clients.
- Distribution Node:
  - SDDF Tunnel: Facilitates the transfer of computational tasks and results between the Distribution Node and the Central Node using gRPC.
  - SDDF Client: Volunteer nodes connected to the Distribution SDDF tunnel for getting molecular structures to perform computational tasks.
- Scheduled Services:
  - Molecular Conformation Generator: Generates 3D conformations from molecular SMILES using either RDKit implementation of the ETKDGv3 algorithm^14^, or Openbabel^33^, optionally optimizing generated conformations using MMFF94 force field.
  - ML-Based Force Field Conformation Generator: Generates new conformations by running molecular dynamics starting from some existing conformation. Instead of traditional force-fields it uses negated gradients of our energy predicting models with respect to coordinates as atomistic forces.
  - AI Enhanced Task Selector: Selects from the generated set of conformations the ones which are challenging for our existing models and pushes them to the task queue. The web server is a FastAPI-based leaderboard system designed to track and display volunteer contributions. It uses a MongoDB database for storing and retrieving user data and contributions.

To handle task queuing and distribution, the platform uses a file-based message broker. This approach leverages external volumes for storage, ensuring reliability and persistence of messages. A primary benefit of this design is avoiding the overhead of managing third-party services, thereby contributing to system uptime and simplifying maintenance. Comparative analyses with third-party message brokers like RabbitMQ and Apache Kafka demonstrate that while these services offer robust features, they also come with additional management overhead. The file-based message broker provides a lightweight, reliable alternative suitable for environments prioritizing uptime and simplicity. gRPC is utilized for communication between different nodes in the system.

The SDDF Client computations are CPU-only, and can be run on architectures supported by the Psi4 library for quantum chemistry calculations.

### Smart distributed data factory ensemble model selection and simulation strategies

#### Initial labeled data

SDDF relies on a relatively small starting dataset of labeled examples, which are used to train its ensemble’s initial models. For the conformational energy prediction task, we obtained our initial dataset by calculating energy for around 800k molecular conformations using DFT. The molecular conformations were generated from ENAMINE molecules, using Python’s RDKit toolkit with different random seeds. DFT calculations were performed through Python’s Psi4 toolkit, using ωB97x density functional and the 6–31 G(d) basis set.

#### Train, validation, and test split

From the initial labeled data we obtained training, validation, and test sets using the approach below:

1. We randomly selected 80% of the molecules as the training set.
2. From the other 20% we selected as the validation set the molecules that had 70% or higher scaffold similarity to any training molecule.
3. The remaining molecules made the test set.

This approach generates splits that meet the following criteria:

1. No matching molecules between the sets.
2. The maximum scaffold similarity between any pair of molecules in the training and test sets should be lower than 70%.

The generated test set contains nearly 25k conformations with over 6k unique scaffolds that do not overlap with the train set examples.

#### Data stream simulation setup

For SDDF evaluation we simulate a setup of active learning on unlabeled data stream. Based on the train set, we initialize 4 sets of examples:

- Pool: the set of examples, from which data stream is simulated.
- Buffer: the set of examples which are ranked to select labeling candidates.
- Seed: the set of examples for training the ensemble’s initial models.
- SDDF: the continuously growing set of examples selected via SDDF algorithm that have corresponding DFT calculations.

**Fig. 6:**
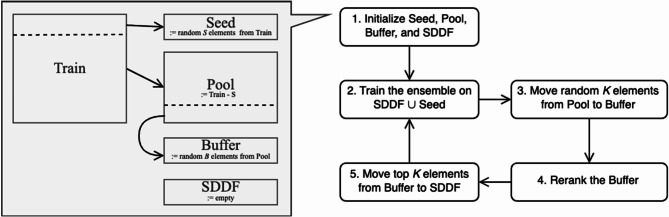
Smart distributed data factory simulation setup. “Pool” denotes the set of examples, from which the data stream is simulated. “Buffer” denotes the set of examples which are ranked to select labeling candidates. “Seed” is the set of examples for training the ensemble’s initial models. “SDDF” denotes the continuously growing set of examples selected via the SDDF algorithm.

We initialize Seed as 100k randomly picked conformations from the train set. From the rest of the train set, Buffer is initialized with 60k random examples. Then, Pool is populated with the train set examples that were not included in either Seed or Buffer. SDDF is empty at the start of simulation.

The evaluation consists of several data selection rounds. During each round we re-train the ensemble models on the combined Seed and SDDF sets, then use the updated ensemble to re-rank Buffer examples based on a disagreement metric, and move the top-ranked 30k examples from Buffer to SDDF. Finally, we re-populate Buffer with 30k random examples from Pool (these examples are removed from Pool). The process is illustrated in Fig. 6. To reduce the impact of instability caused by random initialization in the GCNN models’ performance and to obtain a reliable estimate of the data selection strategies’ effectiveness, we ran the simulations using different random seeds and averaged the results.

#### Ensemble model selection

In order to select appropriate neural network architectures for use in the SDDF ensemble, we studied the PyTorch Geometric implementations of more than 40 GCNN and Point Cloud model architectures from prominent ML conference papers. Specifically, we assessed the ability of methods to incorporate structure information based on the the presence of at least one of the following features:

I. Usage of edge weights during message passing. This would allow us to incorporate the molecular conformation information by using distance-dependent weighting for messages.
II. Usage of edge embeddings during message passing. We can encode atom pair distance and other 3d information into edge embeddings.
III. Usage of atom pair position difference. Position difference is an input feature in some message passing networks, and some attention-based models use it during attention calculations. Thus, the predictions of these models are also conformation-dependent.

We picked 33 appropriate candidates and separately trained each candidate architecture on our train set and evaluated on the validation dataset, using the Adam optimizer and Mean Absolute Error as the optimization loss and validation metric. The details of input features and training hyperparameters are provided in the Supplementary B.

### Molecular dynamics settings

When evaluating the effectiveness of adding MD generated conformations to our datasets, we created train and test examples. For generating training data, the MD simulation for each starting conformation was run for up to 1000 steps (1 picosecond), and every 10th step was saved to a trajectory file. For each starting conformation at most 6 new conformations were selected with lowest values of the ensemble’s confidence score for the predicted forces. As an early stopping criterion, we set a constraint on the system temperature, approximately calculated from the system’s kinetic energy and the number of degrees of freedom.

The MD simulation for the test set of this experiment was run for at most 100 steps (0.1 picoseconds) and each step of the simulation was saved to a trajectory file. The early stopping criterion was applied here too, and the upper bound for temperature during simulation was chosen as 600 K. This was done in order to prevent the system from breaking upon the simulation start, as velocities are initialized randomly.

## Electronic supplementary material

Below is the link to the electronic supplementary material.

Supplementary Material 1

## Data Availability

The energy datasets generated during the current study are available in the Zenodo repository “SDDF Energy Dataset”, 10.5281/zenodo.14008357. Other datasets analyzed during the current study:- ANI-1 is available in the Figshare repository “ANI-1: A data set of 20 M off-equilibrium DFT calculations for organic molecules”, 10.6084/m9.figshare.c.3846712.v1. ANI-2x is available in the Zenodo repository “ANI-2x Release”, 10.5281/zenodo.10108942. NablaDFT analysis was performed using the summary file provided in the GitHub repository’s README.md file (https://github.com/AIRI-Institute/nablaDFT/blob/1.0/README.md). Persistent web link to the summary file: a002dlils-kadurin-nabladft.obs.ru-moscow-1.hc.sbercloud.ru/data/nablaDFT/summary.csv.- NablaDFT 2.0 analysis was performed using the summary file provided in the GitHub repository’s README.md file (https://github.com/AIRI-Institute/nablaDFT/blob/main/README.md). Persistent web link to the summary file: a002dlils-kadurin-nabladft.obs.ru-moscow-1.hc.sbercloud.ru/data/nablaDFTv2/summary.csv.gz.- GEOM is available in the Harvard Dataverse repository “GEOM”, 10.7910/DVN/JNGTDF. QM9 is available in the Figshare repository “Quantum chemistry structures and properties of 134 kilo molecules”, 10.6084/m9.figshare.c.978904.v5. MD17 and MD22 datasets are available in the sGDML.org webpage, https://www.sgdml.org/#datasets.
