# Supplementary material for: Smart distributed data factory volunteer computing platform for active learning-driven molecular data acquisition
Source: Sci Rep. 2025 Feb 28;15:7122. doi: 10.1038/s41598-025-90981-6 (PMC11868574; doi:10.1038/s41598-025-90981-6)
Supplement: Supplementary file 1 — Supplementary Material 1 [file 41598_2025_90981_MOESM1_ESM.pdf]

Supplementary Materials for "Smart Distributed Data Factory Volunteer Computing Platform for Active Learning-Driven Molecular Data Acquisition"

Tsolak Ghukasyan[1,†] **Vahagn Altunyan**[2,†] **Aram Bughdaryan**[3] **Tigran Aghajanyan**[4]

Khachik Smbatyan[5] **Garegin A. Papoian**[6] **Garik Petrosyan**[7,*]

gpetrosyan@deeporigin.com

Deep Origin

[1,2,3,4,5,7]Armenia, [6]USA. † Equal contribution. *Corresponding author

Supplementary A. Related work **2**
Chemical space sampling for dataset creation 2
Volunteer computing 2
Conformational energy datasets 3
Conformational energy prediction with machine learning 4
Supplementary B. Smart Distributed Data Factory platform **5**
Platform implementation details and features 5
Ensemble of machine learning models for active learning 5
Ensemble model selection 5
Node and edge representations 6
Node and edge feature representation alternatives 9
Model training hyperparameters 10
SDDF energy prediction models' performance relative to other models 11
Evaluation of conformation sampling strategies 12
Analysis of the loss predictors' performance 12
Analysis of sampled molecules' distributions 13
Analysis of the computational cost of different DFT methods 15
Supplementary C. Energy prediction dataset and benchmark **17**

Supplementary A. Related work

The use of machine learning (ML) techniques for predicting molecular properties, including conformational energies, has been an active area of research in recent years. One of the key challenges in developing accurate ML models for molecular modeling applications is the availability of high-quality training data.

Chemical space sampling for dataset creation

Traditional approaches to building datasets often rely on brute-force sampling or random selection of molecules, which can be computationally expensive and may fail to capture the most informative instances. To address this issue, several researchers have explored various active learning strategies for intelligent data acquisition.

- Artrith and Behler et al. (2012) [34] performed a neural network-based MD simulation and then used another network to predict the potential energies of the generated trajectory's conformations. They then used DFT to re-calculate the potential energies for the conformations where the predictions of the networks differed from each other, and used these conformations with the newly calculated energies as training examples in the future.
- Zhang et al. (2019) [25] presented the deep potential generator DP-GEN, based on an ensemble of Deep Potential models. They used the maximum standard deviation of predicted atomic forces as the criteria for selecting examples for DFT labeling.
- Smith et al. (2018) [24] proposed using an active learning framework based on an ensemble of ANI models for the creation of a labeled conformational energy dataset, emphasizing that "Less is more" when it comes to dataset size versus quality. In their setup, they iteratively select and calculate potential energies for samples from a given fixed set of conformations until the performance of the ensemble reaches the pre-defined threshold.
- Jung et al. (2024) [35] also used an ensemble of ANI models for active learning-based data sampling, additionally using similarity checks for select unique configurations and augmenting data by adding random noise displacement at each active learning iteration.

Our work builds upon these previous efforts but has several key differences:

1. As the source for molecules for the datasets we use ENAMINE, which is an essential database for drug discovery projects.
2. We performed a comprehensive evaluation of graph neural-network based models and selected an ensemble of distinct models with different architectures. The heterogeneous ensemble approach helps to mitigate the biases of individual models and improves the overall performance of the data sampling process.
3. We performed an evaluation of active learning-based sampling methods and selected the best performing method based on ML models
4. We employ molecular dynamics to further enrich the set of molecular conformations.
5. We introduce a volunteer computing platform, distributing selected sets of molecule conformations to volunteers to perform the calculations on their machines.

Volunteer computing

In creating our system, which uses volunteer computing to analyze molecular properties via QM, we reviewed various distributed computing platforms. Although each provided valuable insights, none completely suited our project's unique needs, especially the need for AI-enhanced datapoint sampling, prompting us to develop a new platform.

- Globus Toolkit (Foster and Kesselman, 1997) [36] and Grid Services for Distributed Systems Integration (Foster et al., 2002) [37] have pioneered in providing secure, high-performance distributed computing infrastructures and service-oriented architectures, respectively. These frameworks excel in academic and institutional settings but lack the flexibility and accessibility needed for engaging a wide, public audience in computationally intensive DFT calculations.
- SETI@home (Anderson et al., 2002) [38], Cosm [39], JXTA [40][41], and XtremWeb (Fedak et al., 2001) [42] demonstrated the potential of public-resource and peer-to-peer computing for tackling large-scale

scientific problems through volunteer participation. However, these platforms are either too specialized (SETI@home) or not optimized (Cosm, JXTA, XtremWeb) for the specific, high-precision computational tasks required in chemistry, such as DFT analysis.

- Entropia (Chien et al., 2003) [43] offered utilizing idle computing resources within more controlled environments, such as corporate networks or through commercial platforms. While they showcased the viability of distributed computing for scientific and commercial applications, their models do not align with our vision of an open, collaborative, and freely accessible platform designed for the global scientific community and public volunteers.
- BOINC (Anderson, 2004) [10][11] stands out for its framework supporting volunteer computing across various scientific disciplines, closely aligning with our objective of widespread public engagement. There have been several usages of BOINC for biomedical research purposes, such as GPUGRID.net [44] for all-atom biomolecular simulations and Folding@home [12] dedicated to understanding protein folding. However, its general-purpose nature requires considerable adaptation to meet the nuanced demands of machine learning model training and inference, molecular property computations using DFT, including task specificity, computational efficiency, and the management of complex scientific data.

Consequently, while acknowledging the foundational work of these predecessors, the unique challenges of our project—namely, the need for a highly flexible, accessible, and scientifically rigorous AI enhanced computing platform—necessitate the development of a solution tailored to the dynamic and precise requirements of molecular simulations and DFT calculations.

Conformational energy datasets

Several publicly available datasets contain energy annotations for molecular conformations, but each presents its own limitations or drawbacks:

- ANI-1 [4] dataset is based on a subset of the GDB-11 database. It contains DFT-calculated energies for approximately 20 million conformations of small organic molecules. However, it is limited to molecules with only H, C, N, and O atom types, with a maximum of 8 heavy atoms (C, N, O). This restricted chemical space may not be representative of the diverse molecular structures encountered in drug discovery or material science applications.
- ANI-2x [31], a more extensive and diverse follow-up dataset to ANI-1, this dataset provides DFT properties at 5 different levels of theory for small organic molecules containing H, C, N, O, S, F, and Cl atom types. While large in the number of molecules and inclusive of more atom types, this dataset mainly contains relatively small molecules.
- NablaDFT [6]: Introduced by Khrabrov et al. 2022, this large-scale dataset comprises 6 million conformations for 1 million molecular structures with C, N, S, O, F, Cl, Br, and H atom types. Although it provides broader coverage of chemical space compared to ANI-1, the dataset is derived from a subset of the MOSES dataset, which was initially designed for molecular generation tasks and may not be optimized for conformational analysis or energy prediction. The conformations in NablaDFT were generated using RDKit [14], without employing molecular dynamics (MD) or sophisticated conformation sampling algorithms. In their recent follow-up work, NablaDFT 2.0 [45], they doubled the number of molecules and conformers in the dataset, and also released approximately 3 million conformations obtained from the relaxation trajectories for around 60,000 examples.
- GEOM [46] dataset contains 37M energy-annotated molecular conformations for 133,000 different molecules from QM9 and 317,000 molecules from experimental data related to biophysics, physiology, and physical chemistry. The average number of heavy atoms in the molecules is below 20 (although if we exclude QM9 molecules, the average rises to over 25, which is a bit higher than NablaDFT), and the level of theory used for DFT calculations is considered by some [45] to be relatively less accurate than the theory level of ANI and NablaDFT datasets.

- MPCONF196 [7]: This benchmark dataset focuses on accurate conformational energies of smaller peptides and medium-sized macrocycles. While valuable for its specific domain, the dataset may not be representative of the broader chemical space relevant to drug discovery or material science applications.
- QM9 [2]: Created by researchers at the University of Warwick, the QM9 dataset consists of geometric, energetic, and electronic properties for a subset of 133,885 stable and synthetically accessible organic molecules, comprising up to 9 heavy atoms (C, N, O, F). While widely used for benchmarking, the dataset's coverage is limited to a specific range of molecular sizes and atom types. Additionally, as noted in the introduction, QM9 provides at most one conformation per molecule, and its overall ratio of unique scaffolds to total examples is less than 2%. Moreover, more than 99% of its test set scaffolds are also present in the train set, indicating leakage.
- QMugs [47]: This dataset, developed by researchers at the University of Cambridge, contains quantum mechanical properties, including energies, for a diverse set of molecular structures. However, the specific details regarding its composition, diversity, and potential biases for conformational analysis tasks are not widely documented or evaluated in the literature.
- Transition1x [48]: This dataset contains 9.6 million DFT calculations of forces and energies of molecular configurations on and around reaction pathways.
- MD17 [49]: The dataset is a collection of over 3.5 million MD trajectories for 8 small organic molecules. MD22 [50], regarded as the next generation of MD17, contains over 200,000 MD-simulated conformations for 7 systems with 42 to 370 atoms.

In our datasets we include molecules from the ENAMINE database, with C, N, S, O, F, Cl, Br, and H atom types. We generate conformations via RDKit and additionally obtain new conformations using MD. We also provide a train and test benchmark with a strict split: a scaffold split, followed by additional fingerprint similarity-based filtering. For example, the NablaDFT papers do not perform the additional similarity filtering step. It is important to follow such a strict split rule to avoid over-optimistic evaluation results for models. We specify the training set examples to make the evaluation of different model architectures fair. It should be mentioned that there are other benchmark datasets for related tasks. For example, TorsionNet500 [66] provides a test set of 500 molecule fragments with DFT torsion profiles (12k DFT-optimized geometries and energies).

Conformational energy prediction with machine learning

In recent years, several neural network architectures have emerged which allow the creation of conformation aware models. It is fundamental for all methods to be invariant to translations and rotations of the input molecule's coordinates. ANI-1 model and its extensions [5][31][32] use multi-layer perceptrons and single-atom atomic environment vectors as input for neural networks-based energy and force prediction in molecular systems. Graph convolutional neural networks (GCNN) have been a particularly popular architecture for molecular property prediction tasks [51] and still demonstrate competitive results on several benchmarks [52][53][54][55][67]. AIMNet2 [68] and MACE-OFF23 [69], two recent notable models for energy prediction, also utilize GCNN-inspired message-passing architectures. Transformers [56] have also shown great capacity to encode 3D structural information via adapted attention mechanisms [57], and have been adopted in several molecular property prediction models [58]. More recently, SE(3)-equivariant Transformers have gained popularity [59][60][61][62], showing state-of-the-art results on some benchmarks.

Supplementary B. Smart Distributed Data Factory platform

This section describes the capabilities and implementation of our volunteer computing platform in more detail, including the ensemble of machine learning learning for active learning-based data sampling, their architecture and training hyperparameters.

Platform implementation details and features

gRPC is utilized for communication between different nodes in the system. gRPC offers several advantages, including:

- High Performance: gRPC uses HTTP/2 for transport, providing efficient binary serialization and reducing latency. Benchmarks [63] indicate that gRPC can achieve significantly better performance in terms of latency and throughput compared to traditional REST APIs. For example, gRPC handled requests almost seven times faster than REST in a benchmark test, with gRPC achieving 141 requests per second compared to REST's 22.9 requests per second.
- Scalability: gRPC supports multiple concurrent connections and efficient load balancing, crucial for the system's scalability. Google's internal use of gRPC demonstrates its capability to handle large-scale, distributed systems efficiently.
- Reliability: gRPC's strong typing and contract-first approach ensure reliable and well-defined communications between nodes, reducing the risk of errors. It includes built-in support for automatic retries, backoff strategies, and deadlines/timeouts, enhancing the reliability of communications.

In order to verify the calculation results and also to prevent the malicious use of the platform by a single user or a group of users, the submissions by all users are continuously screened. Upon registration, each user first receives several tasks, for which the computation results are already known. The submitted results are checked against the known results, and if they do not match for 80% of the cases, the user is not sent further tasks. After the initial screening procedure, the submissions of the user are continued to be verified at a rate of 1 per 100 tasks on average.

The platform's website also provides some community features such as a public leaderboard of top-ranked contributors. The ranking is based on the estimated amount of total calculations by each user. Visitors can also apply different filters on the leaderboard and, for example, view the leaderboard for each project.

Ensemble of machine learning models for active learning

In this section, we provide a detailed explanation of our ensemble model selection process and the performance of the machine learning models used for active learning and energy prediction tasks. The ensemble approach combines various graph convolutional neural networks (GCNNs) models, each with different ways of aggregating messages and applying attention. We explain the structure and function of the selected models GeneralConv, PNAConv, GENConv, TransformerConv, and ResGatedGraphConv. To improve accuracy, we used node and edge representations, including Point Pair Features (PPF), which helped boost the performance of molecular energy predictions. We also compare our results with leading models like ANI-2x and GemNet, focusing on how they perform with molecules containing bromine atoms. The results show that even without using ensembles, many of our individual models outperform ANI-2x. The following sections contain analysis on how our models performed during training, validation, and ranking tasks, along with a look at the distribution of molecules selected in an active learning simulation.

Ensemble model selection

We trained and evaluated 33 different neural network models implemented in PyTorch Geometric [16] for predicting conformational energy. From these evaluations (Table S1), we chose the top five models with the best MAE scores on the validation set: GeneralConv [17], PNAConv [18], GENConv [19], TransformerConv [20], and ResGatedGraphConv [21], all implemented in PyTorch Geometric. We also demonstrated that incorporating Point Pair Features [22] for bonded atoms enhances the performance of the models.

GeneralConv is the implementation of the general GCNN layer from the "Design Space for Graph Neural Networks" [17] paper. We used the mean aggregation scheme for the messages, with only 1 message calculated per atom pair, without linear function in skip connection and without adding attention to message computation. We included the edge embedding in the computation, weighting each embedding based on the distance between the atoms.

PNAConv architecture from the "Principal Neighbourhood Aggregation for Graph Nets" paper [18] extends the traditional GCNN by employing multiple message aggregation functions. The operator first computes several independent aggregations of the messages using their mean, maximum, minimum and standard deviation, then additionally scales these aggregations based on the degree of the message-receiver node:

$$x'_i = \gamma_\Theta(x_i, AGG_{j\in N(i)} h_\Theta(x_i, x_j, e_{ij}))$$

where x_i is the embedding vector of the i-th node, e_{ij} is the embedding of the edge between i-th and j-th nodes, $N(i)$ returns the neighbors of i-th node, h_Θ and γ_Θ are MLPs. AGG is the PNAConv aggregation applied to the output of h_Θ and is defined as follows:

$$AGG = [1, S(d, \alpha = 1), S(d, \alpha =- 1)] \otimes [\mu, \sigma, max, min]$$

where $\otimes$ is the tensor product, d is the degree of the node, $S(d, \alpha) = (\frac{log(d+1)}{\delta})^{\alpha}$, and δ is a normalization parameter computed over the training set.

GENConv implements the Generalized Graph Convolution (GENConv) from the "DeeperGCN: All You Need to Train Deeper GCNs" [19] paper. It proposes using aggregation functions that unify the properties of multiple basic aggregations (for example, softmax to unify mean and max). In our work, we used softmax to aggregate the node messages:

$$x'_i = \gamma_\Theta(x_i + Softmax_{j\in N(i)} ReLU(x_j + e_{ij}))$$

The notation of x_i , e_{ij}, $N(i)$, γ_Θ is the same as in PNAConv.

TransformerConv is from "Masked Label Prediction: Unified Message Passing Model for Semi-Supervised Classification" paper [19]. It computes multi-head attention on neighbor node embeddings, aggregates the attention head outputs independently and uses their concatenation as the message:

$$x'_i = W_1 x_i + \sum_{j\in N(i)} \alpha_{ij}(W_v x_j + W_e e_{ij})$$

where W_i is a weights matrix, and α_{ij} are the attention coefficients obtained via attention (d is the size of embeddings x_i):

$$\alpha_{ij} = softmax(\frac{(W_q x_i)^T (W_k x_j)}{\sqrt{d}} + W_e e_{ij})$$

ResGatedGraphConv implements the residual gated graph convolutional operator from the "Residual Gated Graph ConvNets" paper [21]:

$$x'_i = W_1 x_i + \sum_{j\in N(i)} \eta_{ij} \odot (W_2[x_j \,||\, e_{ij}])$$

where || is the concatenation operator, $\odot$ is the Hadamard pointwise multiplication operator, W_i is a weights matrix, and $\eta_{ij} = \sigma(W_3[x_i||e_{ij}] + W_4[x_j||e_{ij}])$, with σ denoting the sigmoid function.

Node and edge representations

We construct the input graph with the atoms as the nodes and the edges of the graph are defined based on the distance between atoms or the presence of a bond. If atoms *i* and *j* had distance below 4Å or had a chemical bond, we added an edge in the graph between the corresponding nodes.

In our ensemble models, each node feature is a trainable embedding for the corresponding atom type. We additionally tested pre-trained Uni-Mol node features [64], which also showed success in tasks that rely on

molecular 3D properties. However, Uni-Mol employs parameter-heavy Transformer layers and significantly slowed down the inference time, while yielding very small or no gains for the selected models.

We encode each edge using the concatenation of 3 feature sets:

1. Embedding for each unique atom pair (e.g., “H-H”, “C-H”, or “O-H”).
2. Embedding for each edge type. We used 7 different edge types: 6 types indicating the bond (SINGLE, DOUBLE, TRIPLE, AROMATIC, IONIC, HYDROGEN), and another UNSPECIFIED type when the node atoms do not have a bond, but their inter-atom distance is lower than 4Å.
3. Expanded version of rotation-invariant Point Pair Features (PPF):
    a. distance between source and receiver nodes ($||d_{ji}||$, where d_{ji} denotes the difference vector between points)
    b. angle between d_{ji} and the surface normal vector of node i
    c. angle between d_{ji} and the surface normal vector of node j
    d. angle between the surface normal vectors of nodes i and j

    We expanded the distance and angles to a 16-dimensional embedding, using 8-class one-hot encoding based on evenly spaced boundaries for each feature and a linear layer on top of each.

The performance of explored input features alternatives is provided in the following section.

Table S1. Performance evaluation of GCNN and Point Cloud models on the validation set. MAE is reported in kcal x mol^{-1}.

**Model**	**Validation MAE**
GENConv	2.65
PNAConv	2.85
TransformerConv	3.38
ResGatedGraphConv	3.47
GeneralConv	5.00
AntiSymmetricConv	5.11
GPSConv	5.85
PPFConv	5.85
CGConv	6.42
PointNetConv	7.04
RGATConv	7.29
HEATConv	7.84
GATv2Conv	8.30
SplineConv	8.89
GMMConv	9.25
GatedGraphConv	9.56
NNConv	11.84
XConv	11.91
PointTransformerConv	14.24
PDNConv	14.44
ARMAConv	15.07
GraphConv	19.31
LEConv	19.50
ChebConv	19.56
PointGNNConv	19.72
TAGConv	22.68
MixHopConv	24.12
GCNConv	26.17
GCN2Conv	27.59
WLConvContinuous	27.81
LGConv	28.33
FAConv	28.57
APPNP	29.35

Node and edge feature representation alternatives

For the selected 5 models we attempted to enrich their edge representations using PPF. The evaluation on the validation set demonstrated that these features consistently improved the MAE score (Table S2). Therefore, in all following experiments we used them as part of the models.

Table S2. Validation results for different edge representations.

Model	Edge representation			
	Edge type + Edge distance		Edge type + Edge distance + PPF	
	Valid RMSE	Valid MAE	Valid RMSE	Valid MAE
ResGatedGraphConv	5.65	3.47	**4.3**	**2.38**
GENConv	5.11	2.96	**3.65**	**1.87**
GeneralConv	8.34	5.00	**6.96**	**3.72**
TransformerConv	5.46	3.38	**5.01**	**2.24**
PNAConv	**4.37**	2.85	4.81	**2.23**
Average	*5.79*	*3.53*	***4.95***	***2.49***

Table S3. Validation results for different node representations.

Model	Node representation					
	Atom type		Atom type + Uni-Mol		Uni-Mol	
	Valid RMSE	Valid MAE	Valid RMSE	Valid MAE	Valid RMSE	Valid MAE
ResGatedGraphConv	**4.3**	**2.38**	5.64	2.73	6.04	2.73
GENConv	**3.65**	**1.87**	4.73	**1.87**	4.24	1.92
GeneralConv	**6.96**	3.72	8.77	**2.62**	11.46	2.72
TransformerConv	**5.01**	**2.24**	5.48	3.14	7.97	2.98
PNAConv	4.81	2.23	**3.99**	**2.12**	12.25	3.76
Average	***4.95***	***2.49***	*5.72*	*2.50*	*8.39*	*2.82*

With the aim of finding more efficient feature representations, we additionally trained the models with Uni-Mol node features. We tested 3 feature configurations: (i) only trainable atom type embeddings, (ii) Uni-Mol features and trainable atom type embeddings, (iii) only Uni-Mol as node features. In both configurations, Uni-Mol embeddings were frozen during training. The addition of Uni-Mol embeddings did not improve ResGatedGraphConv, GENConv, TransformerConv models, and only showed slight MAE improvements for GeneralConv and PNAConv models (Table S3). During the training, we observed that Uni-Mol-based models started off relatively well on validation metrics, but then plateaued more quickly than the models without them (Fig. S1). Thus, we decided not to use Uni-Mol in further experiments as it significantly increased the inference time and memory requirements without obvious improvement in validation metrics.

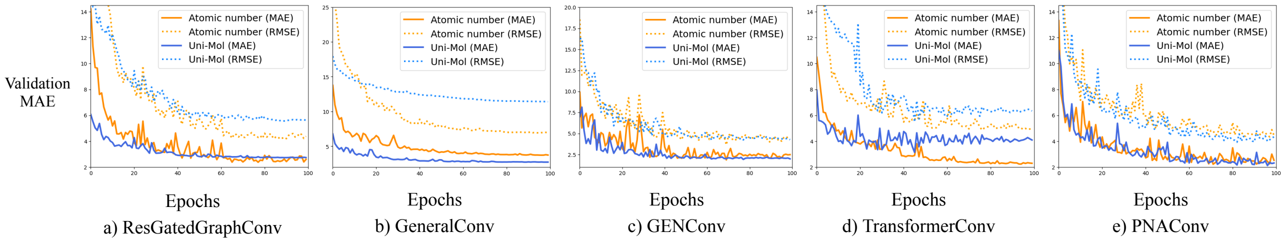

Figure S1. Impact of node feature selection on model training.

PPF edge representations rely on the surface normal of the atoms as the anchor vector to calculate the angles. To avoid the relatively expensive computation of the normals, we additionally tested an alternative way of PPF calculation where the anchor vector for each atom is the normalized sum of the differences from its neighboring atoms' position (Table S4). We refer to the original PPF as PPF-Normal hereafter, and the difference vector-based alternative is denoted as PPF-Diff. SDDF models based on each variant of PPF are respectively called SDDF-Normal and SDDF-Diff.

Table S4. Validation results for different PPF feature anchor vector calculation methods.

Model	Edge representation			
	PPF-Normal		PPF-Diff	
	Valid RMSE	Valid MAE	Valid RMSE	Valid MAE
ResGatedGraphConv	4.3	2.38	**2.70**	**1.87**
GENConv	3.65	1.87	**2.52**	**1.76**
GeneralConv	6.96	**3.72**	**5.63**	3.80
TransformerConv	**5.01**	**2.24**	6.59	4.51
PNAConv	4.81	2.23	**2.49**	**1.75**
Average	*4.95*	***2.49***	***3.99***	*2.74*

The final models that were presented in the main manuscript use PPF-Diff version of the features.

Model training hyperparameters

For the initial model selection procedure, we trained each model for up to 100 epochs, using Mean Absolute Error loss function and Adam optimizer with 1e-4 initial learning rate. In the experiment for the evaluation of SDDF sampling strategies, we trained the models for up to 200 epochs. For the selected 5 architectures, we trained their final versions for up to 500 epochs. In each training run, the best checkpoint was selected based on the MAE score on the validation set. For regularization we use dropout with 0.15 probability. Mini-batch size is 128, where each sample is a graph for a single conformation. Unless specified otherwise, at the initial model selection stage we used the models' default parameters and did not perform thorough hyperparameter tuning for each model separately.

We trained the models with targets represented in Hartree units. For all training runs, we shifted each molecule's target energy by subtracting the estimated self-interaction atomic energies. The self-interaction atomic energies of each atom type were estimated via a linear regression model, where the atom type counts of a molecule are the input features and its total energy is the target. During the active learning simulation experiments, the estimated energies were re-calculated before every simulation round using the available Seed and SDDF sets. For the released energy prediction models that were benchmarked on our test set, we used the following self-interaction energies for each atom type:

- H: -0.60210 E_{h}
- C: -38.10081 E_{h}
- N: -54.73759 E_{h}
- O: -75.20245 E_{h}
- S: -398.13982 E_{h}
- Cl: -460.18514 E_{h}
- Br: -2573.77264 E_{h}
- F: -99.82343 E_{h}

While we trained the models using Hartree units, all evaluation results are reported in kcal x mol^{-1} unless specified otherwise.

SDDF energy prediction models' performance relative to other models

We compared our individual conformational energy prediction models, as well as the ensembles, with the ANI-2x ensemble and the GemNet model trained on NablaDFT. Since ANI-2x was not trained on molecules containing bromine atoms, we present the evaluation results of our models on both the full test set and the subset without bromine-containing molecules (Table S5). Even without ensembling, almost all individual models outperformed ANI-2x on the test set.

Table S5. Comparison of the performance of ANI-2x and SDDF ensemble models on our test set (MAE in brackets indicates the results on the test subset excluding examples with Bromine atoms).

**Model**		**Test RMSE***	**Test MAE***
SDDF-Normal	PNAConv	4.14 (4.18)	2.53 (2.52)
	ResGatedGraphConv	4.25 (4.26)	2.77 (2.75)
	GENConv	4.23 (4.32)	2.28 (2.27)
	GeneralConv	6.90 (6.89)	4.11 (4.09)
	TransformerConv	5.08 (5.16)	2.72 (2.71)
	Ensemble{PNAConv, ResGatedGraphConv, GENConv}	3.78 (3.83)	2.17 (2.16)
	Ensemble{all}	4.13 (4.19)	2.27 (2.26)
SDDF-Diff	PNAConv	3.35 (3.38)	2.06 (2.06)
	ResGatedGraphConv	3.39 (3.42)	2.25 (2.24)
	GENConv	3.58 (3.60)	2.25 (2.23)
	GeneralConv	6.02 (5.97)	4.10 (4.08)
	TransformerConv	7.31 (7.34)	5.12 (5.11)
	Ensemble{PNAConv, ResGatedGraphConv, GENConv}	**2.89 (2.92)**	**1.83 (1.82)**
	Ensemble{all}	3.52 (3.54)	2.31 (2.30)
ANI-2x**		— (5.07)	— (2.84)

*kcal x mol^{-1} (using 627.509 as the conversion coefficient from Hartree units)

**Does not support Bromine

Since direct comparison of models trained on datasets with different DFT theory levels would not be valid (Fig. S2), we decided to compare such models based on their ability to rank conformations using the predicted energy (Table S6). To keep the comparison fair, we did not use in this test any conformation generated by our methods. The SDDF ensemble trained on the SDDF train subset of the full dataset performed slightly better than the ANI-2x ensemble, but worse than NablaDFT in this ranking task. The lower score of the tested SDDF models can be attributed to the fact that the training set contained fewer conformations per molecule on average compared to NablaDFT.

Table S6. Comparison of the performance of ANI-2x, GemNet model trained on NablaDFT, and SDDF ensemble models on our test set (excluding examples with Bromine atoms and molecules with a single conformation) in the per-molecule conformation ranking task. The reported score is the average of Spearman correlation coefficients for each molecule.

**Model**		$\rho_{\mathbf{Spearman}}$
SDDF-Diff	PNAConv	0.9029
	ResGatedGraphConv	0.8920
	GENConv	0.8996
	GeneralConv	0.7662
	TransformerConv	0.7312
	Ensemble{PNAConv, ResGatedGraphConv, GENConv}	0.9177
ANI-2x		0.9120
NablaDFT GemNet-OC_train_large		0.9388

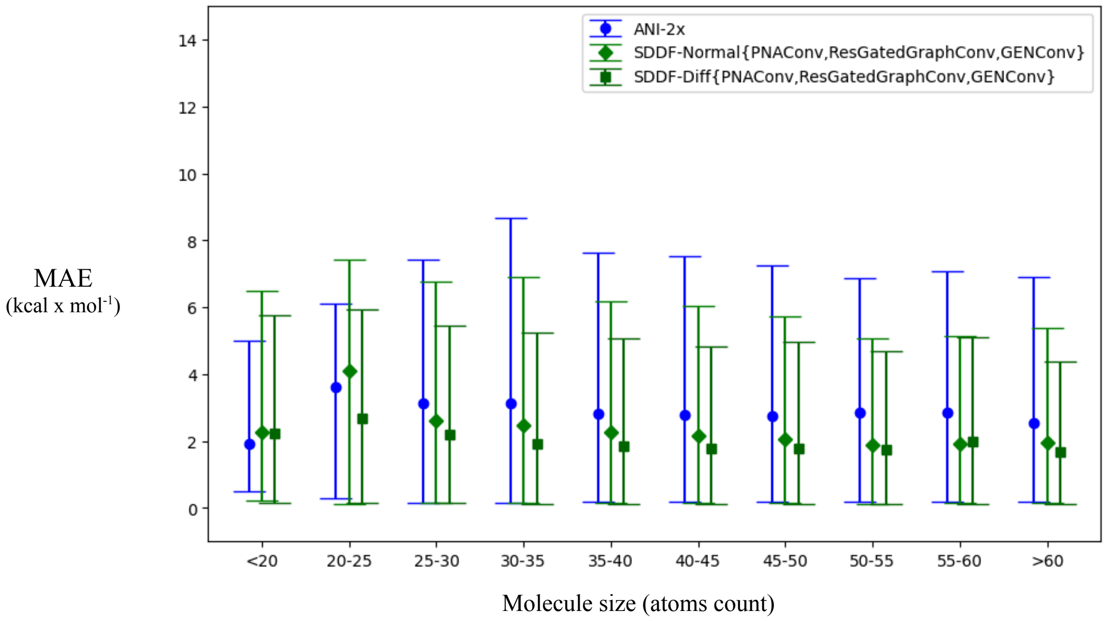

Figure S2. Comparison of models' SDDF test set prediction errors based on molecule size.

Evaluation of conformation sampling strategies

Table S7. Energy prediction test set MAE of the ML ensemble for different conformation sampling strategies.

Sampling strategy	Seed	Step 1	Step 2	Step 3	Step 4
RANDOM	5.72	6.20	4.51	4.52	4.25
x5GENConv	4.26	4.73	4.37	4.35	3.77
SDDF-VAR	5.72	5.21	4.08	4.00	3.87
SDDF-LOSSFN	5.72	4.10	3.97	3.89	3.73

Table S8. Energy prediction test set MAE of the ML ensemble for the random conformation sampling strategy.

Sampling round	Random seed 1	Random seed 2	Average
Step 1	5.1871	7.218	6.20
Step 2	3.8022	5.2263	4.51
Step 3	3.5056	5.5245	4.52
Step 4	3.4217	5.0775	4.25

Table S9. Energy prediction test set MAE of the ML ensemble for the model loss predictor-based conformation sampling strategy.

Sampling round	Random seed 1	Random seed 2	Average
Step 1	4.4335	3.7649	4.10
Step 2	3.8397	4.1047	3.97
Step 3	3.6466	4.1269	3.89
Step 4	3.7079	3.7524	3.73

Table S10. Energy prediction test set MAE of the ML ensemble for the variance-based conformation sampling strategy.

Sampling round	Random seed 1	Random seed 2	Average
Step 1	4.1141	6.2959	5.21
Step 2	3.8152	4.3525	4.08
Step 3	4.0369	3.9711	4.00
Step 4	3.9435	3.7882	3.87

Analysis of the loss predictors' performance

To evaluate the loss prediction models' ability to estimate the energy prediction MAE on out-of-distribution examples, we trained an error predictor on a random subset (100k examples) of the SDDF train set, and calculated the Spearman correlation between the loss predicted by the model and the actual error on the SDDF test set. The SDDF test set is out-of-distribution compared to the train examples because we performed scaffold split first, followed by similarity filtering. Fig. S3 illustrates the distribution of the target error values on the train and test sets used in this evaluation.

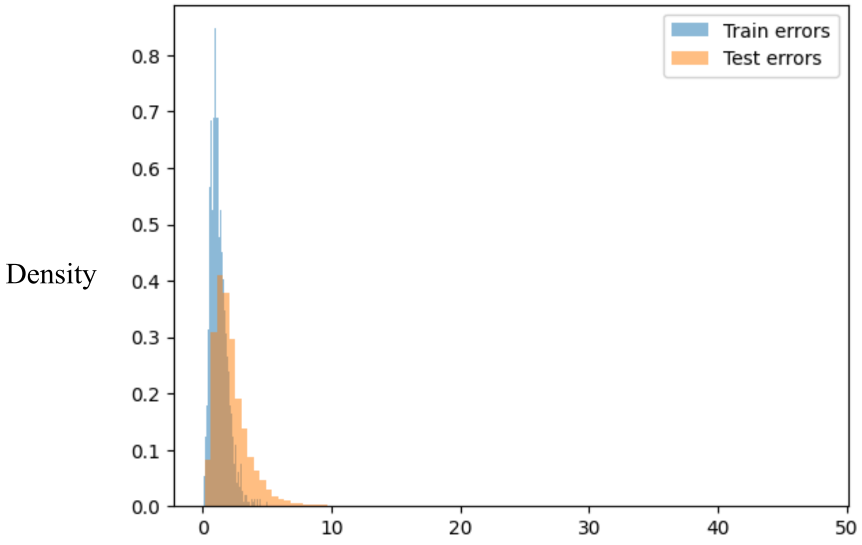

Figure S3. The distribution of target MAE values in the train and test examples.

As shown in Table S11, our loss predictors as an ensemble show 0.39 positive correlation with the actual error on out-of-diastribution data. For comparison, the correlation of the energy prediction error with molecule weight or molecule atom count is much lower at 0.0208 and 0.0127 respectively. We additionally calculated the enrichment factor to verify that the loss predictor ranks the most challenging molecules (defined here as molecules for which the true error is higher than 8 kcal/mol) at the top: the ensemble of loss predictors places nearly 6 times more challenging examples inside the top 1% compared to the random ranking. In addition, we calculate the same metrics for in-distribution data (also shown in Table S11): a random subset of the original SDDF training set that did not have any intersections with the training set of the error predictors.

Table S11. The performance of loss predictors on in-distribution and out-of-distribution examples. The values are averaged over 3 different training runs for each ensemble.

**Loss predictor**	**In-distribution examples**			**Out-of-distribution examples**		
	**MSE**	**Spearman corr.**	**EF@1%**	**MSE**	**Spearman corr.**	**EF@1%**
GENConv	0.6723	0.3206	10.1449	2.5675	0.2722	8.5714
PNAConv	0.7044	0.3615	8.8235	1.9921	0.3078	14.8148
ResGatedConv	0.9648	0.3704	7.7670	1.8613	0.3081	14.1026
GeneralConv	4.4605	0.3403	5.8824	4.9322	0.2772	3.0303
TransformerConv	7.1660	0.2791	3.5616	8.6199	0.2404	3.2051
Ensemble	1.1739	0.4345	6.6667	1.9768	0.3922	5.7692

Analysis of sampled molecules' distributions

We compared the molecules selected by different sampling strategies in the active learning simulation setup. As illustrated in Fig. S4, ensemble-based sampling algorithms do not demonstrate bias towards the selection of relatively large or small molecules and the selected conformations have similar molecule size distribution across all strategies. We also analyzed the distribution of the selected conformations' energies and observed a similar absence of divergence between the different strategies' distributions (Fig. S5).

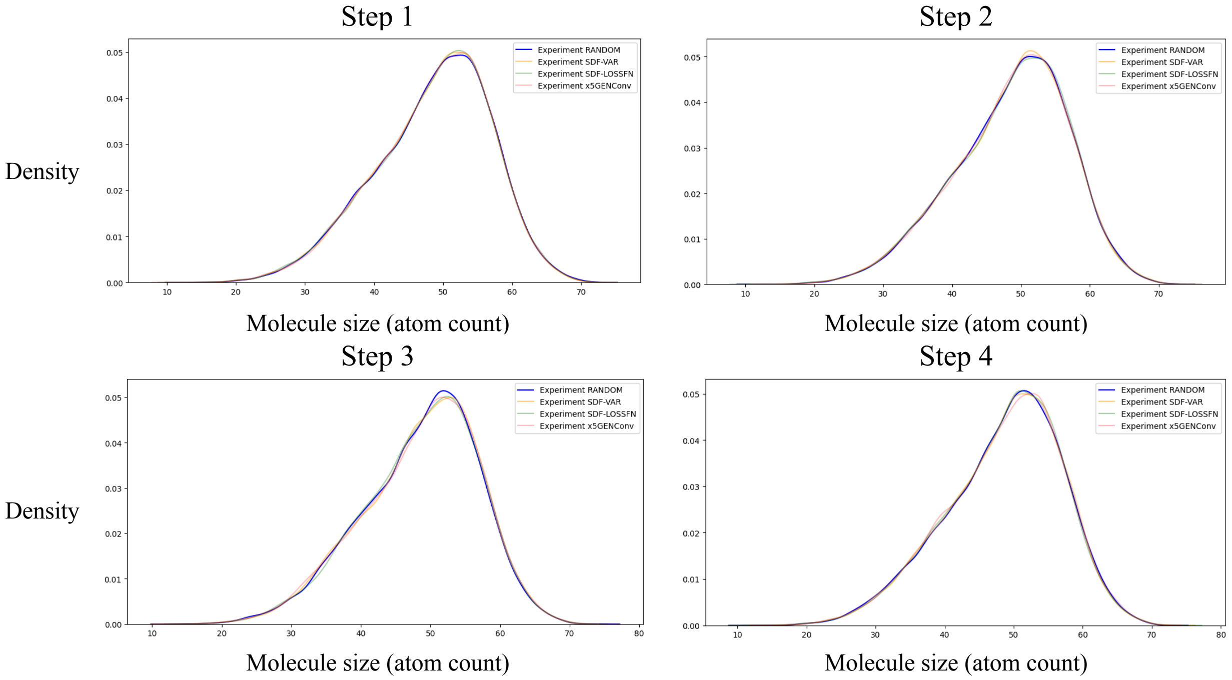

Figure S4. Comparison of molecule sizes for selected conformations at different steps of simulation for the 4 sampling strategies.

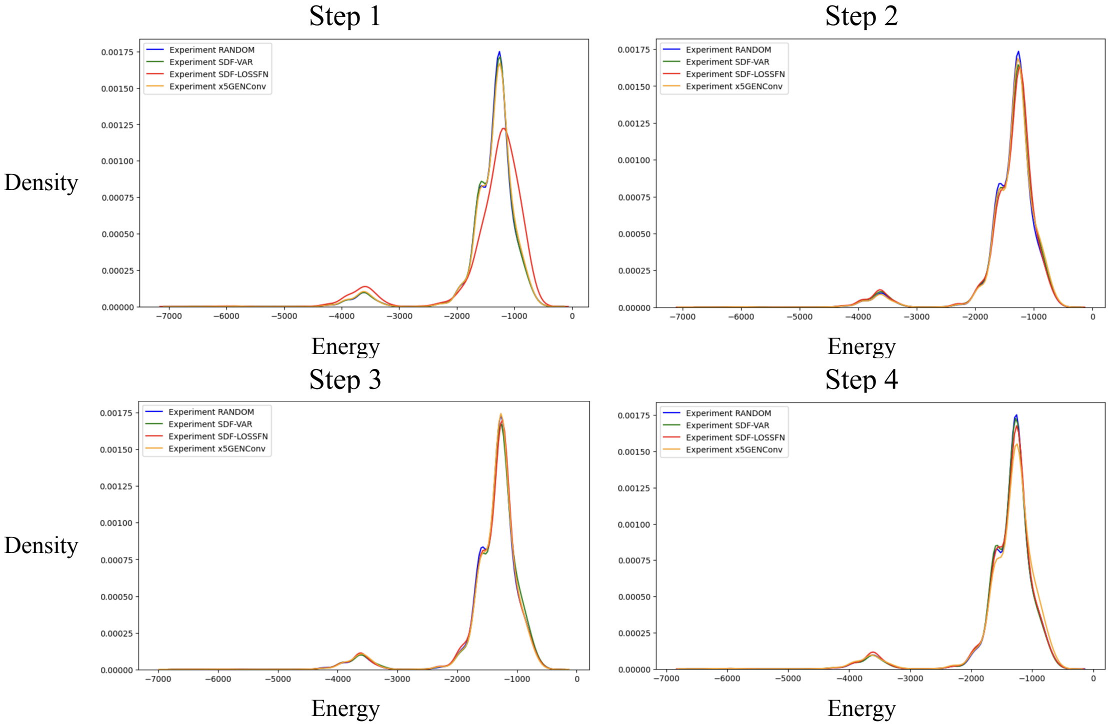

Figure S5. Comparison of energies for selected conformations at different steps of simulation for the 4 sampling strategies.

Analysis of the computational cost of different DFT methods

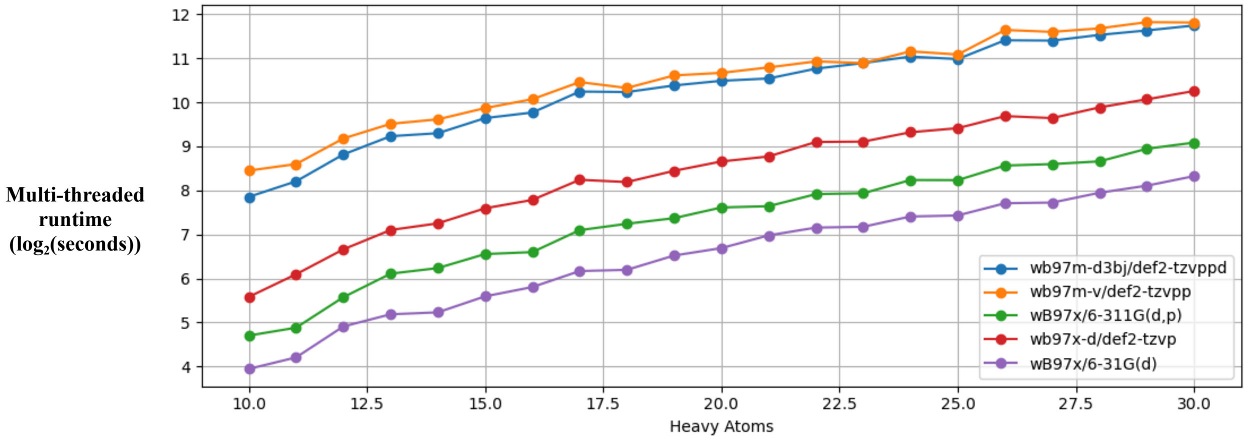

Figure S6. The average conformational energy calculation runtime of different DFT methods for a single molecular conformation based on the number of heavy atoms (multi-threaded regime (3 threads); averaged over different molecules for each heavy atom count).

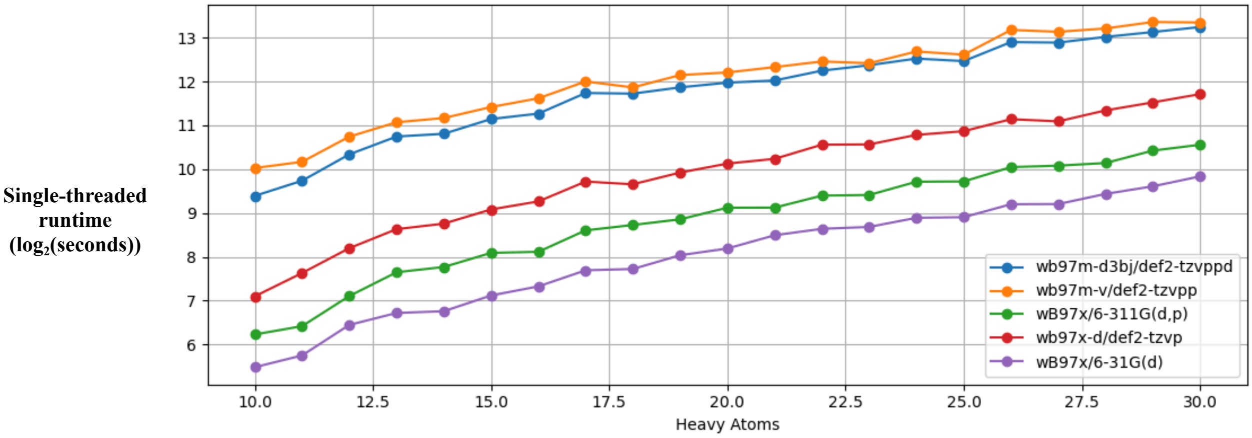

Figure S7. The average conformational energy calculation runtime of different DFT methods for a single molecular conformation based on the number of heavy atoms (single-threaded regime; averaged over different molecules for each heavy atom count).

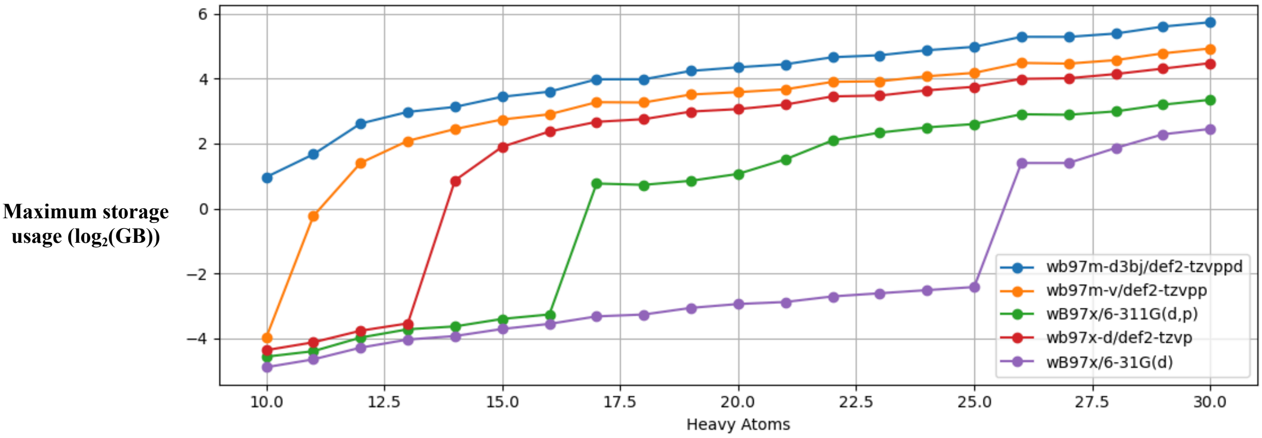

Figure S8. The maximum storage usage during the conformational energy calculation runtime of different DFT methods for a single molecular conformation based on the number of heavy atoms (averaged over different molecules for each heavy atom count).

We performed an analysis of the resource requirements for 5 different DFT theory levels and different molecule groups with heavy atom counts from 10 to 30. The calculations were performed on a machine with an AMD EPYC 9654 processor (96 cores, 2.4 GHz base, 3.7 GHz boost). We also put a limit of 10GB on the RAM usage by Psi4. The results of the calculations are provided in Figures S6, S7, S8. The choice of the appropriate DFT method for a particular dataset creation project is critical, and it should consider the tradeoff between accuracy and the computational cost. Using a highly demanding DFT method might impact the volunteer engagement negatively, as it will exclude many potential volunteers whose hardware does not meet the requirements, as well as slow down the overall labelling process. In SDDF platform's initial dataset creation project we used ωB97X/6-31G(d), however in the future projects of the platform the option of other DFT methods will be added. In the SDDF conformational energy dataset creation project, the choice of ωB97X/6-31G(d) theory level for DFT calculations was based on its use in the ANI datasets and models, and also because we believe it provided a good tradeoff of computational cost against accuracy. The evaluation shows that using alternative DFT methods (for example, with a triple-zeta basis set) significantly increases the runtime and the storage usage requirements (around 2-10 times for molecules with 25 heavy atoms).

Supplementary C. Energy prediction dataset and benchmark

The decision to create a new dataset for the energy prediction task is motivated by the limitations (particularly, the lack of diversity and the presence of train-test leakage) in the existing datasets, mentioned in the related work. As shown in Table S12, our SDDF dataset offers superior scaffold diversity compared to QM9, ANI-1, NablaDFT, and also better conformation diversity compared to QM9. We also believe it is very important to have a dataset based on molecules from a “real-world” database such as ENAMINE, which is one of the most popular compound libraries used in drug discovery projects. The dataset generation is ongoing (another very important factor) and we expect to continually increase the conformational diversity.

Table S12. Diversity in molecular energy datasets. For ANI-2 [65], the scaffold diversity is not determined as the dataset does not provide bond information for its entries.

**Dataset**	**Conformation diversity** (|Conformations| x |SMILES|$^{-1}$)	**Scaffold diversity** (|Scaffolds| x |SMILES|$^{-1}$)
QM9	1.01	0.016
NablaDFT	5.32	0.447
NablaDFT 2.0	8.11	0.232
ANI-1	383.86	0.005
ANI-2x	19.13	-
GEOM	82.22	0.531
MD17	451,389.38	$<10^{-5}$
MD22	31,917.43	$<10^{-4}$
SDDF	5.19	0.752

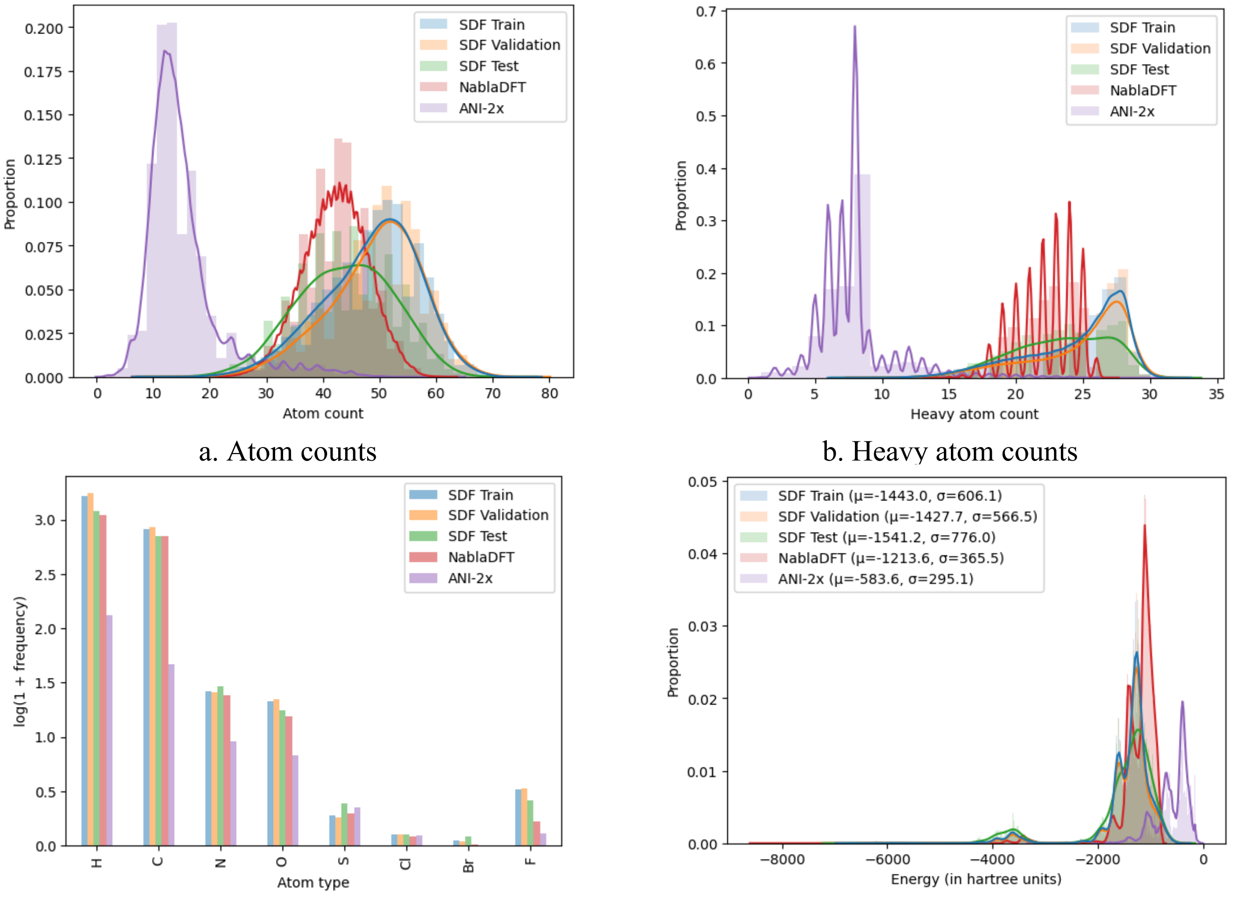

a. Atom counts

b. Heavy atom counts

c. Atom type frequencies

d. Energies (in hartrees)

Figure S9. Comparison of atom count, heavy atom count and atom types distributions in ANI-2x, NablaDFT datasets and SDDF train, validation, test splits.

Our full labeled dataset contains 2,170,553 conformations, including 535,338 that were generated using RDKit, another 1,151,936 that were generated using RDKit and optimized with MMFF94 force field, and 483,279 generated using MD on the RDKit conformations. The statistics for the created SDDF benchmark training, validation and test sets are provided in Table S13.

Table S13. Train, validation, and test datasets size statistics of our benchmark.

**Dataset**	**Molecules**	**Scaffolds**	**Conformations**
SDDF Train	274108	175179	638617
SDDF Validation	58293	38296	134732
SDDF Test	10063	8310	24890

The choice of ωB97X/6-31G(d) theory level for DFT calculations was based on its use in the ANI datasets and models, and also because we believe it provided a good tradeoff of computational cost vs accuracy (section "Analysis of the computational cost of different DFT methods" provides more details). The chosen DFT theory level ωB97X/6-31G(d) has been used in recent studies of conformational energies (such as [70]) and was found to be relatively accurate as reported in [71].
